# Eutrophication and Deoxygenation Forcing of Marginal Marine Organic Carbon Burial During the PETM

**DOI:** 10.1029/2021PA004232

**Published:** 2022-03-03

**Authors:** Nina M. Papadomanolaki, Appy Sluijs, Caroline P. Slomp

**Affiliations:** ^1^ Department of Earth Sciences Faculty of Geosciences Utrecht University Utrecht The Netherlands

**Keywords:** primary productivity, organic carbon, δ^13^C, Paleocene‐Eocene Thermal Maximum, stratification, phosphorus, oxygen, recovery

## Abstract

The Paleocene‐Eocene Thermal Maximum (PETM) is recognized globally by a negative excursion in stable carbon isotope ratios (δ^13^C) in sedimentary records, termed the carbon isotope excursion (CIE). Based on the CIE, the cause, duration, and mechanisms of recovery of the event have been assessed. Here, we focus on the role of increased organic carbon burial on continental margins as a key driver of CO_2_ drawdown and global exogenic δ^13^C during the recovery phase. Using new and previously published sediment proxy data, we show evidence for widespread enhanced primary production, low oxygen waters, and high organic carbon (C_org_) burial in marginal and restricted environments throughout the δ^13^C excursion. With a new biogeochemical box model for deep and marginal environments, we show that increased phosphorus availability and water column stratification on continental margins can explain the increased C_org_ burial during the PETM. Deoxygenation and recycling of phosphorus relative to C_org_ were relatively mild, compared to modern day anoxic marine systems. Our model reproduces the conditions reconstructed by field data, resulting in a burial of 6,000 Pg across the PETM, in excess of late Paleocene burial, and ∼3,300 Pg C for the critical first 40 kyr of the recovery, primarily located on continental margins. This value is consistent with prior data and model estimates (∼2,000–3,000 Pg C). To reproduce global exogenic δ^13^C patterns, this C_org_ burial implies an injection of 5,000–10,000 Pg C during the first ∼100–150 kyr of the PETM, depending on the source's δ^13^C (−11‰ to −55‰).

## Key Points

- Data and modeling show increased productivity, deoxygenation, and organic C burial in marginal and restricted environments during the PETM
- Excess burial of ∼3,300 Pg organic C burial at the early stages of the PETM recovery
- Excess organic C burial during recovery requires several thousands of Pg of ^13^C‐depleted carbon input to match global exogenic δ^13^C trends

## 1 Introduction

**Figure 1:**
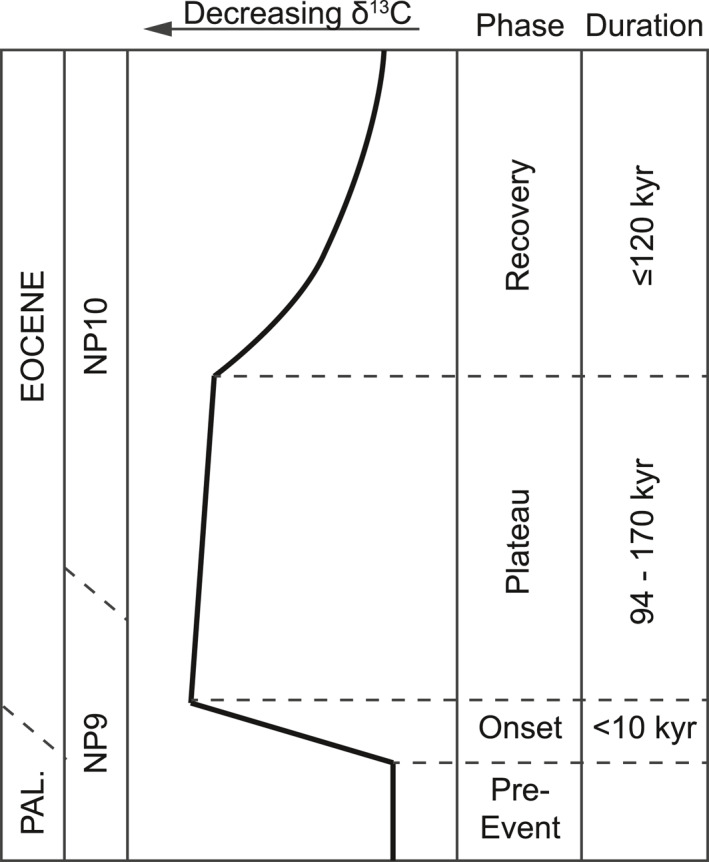
Idealized representation of the carbon isotope excursion (CIE) associated with the PETM. The four key phases of the CIE are shown: the preevent stable background, the δ^13^C decrease across the onset, the stable or slightly increasing values of the “plateau” and the increasing δ^13^C of the recovery. As this δ^13^C curve does not represent a specific site, the boundaries between the Paleocene and Eocene, and Zones NP9 and NP10, are approximate. The duration for the onset (Kirtland Turner, 2018 and references therein), plateau (Zeebe & Lourens, 2019 and references therein), and recovery (e.g., Bowen, 2013; Murphy et al., 2010; Zeebe et al., 2009) of the The Paleocene‐Eocene Thermal Maximum (PETM) are also given.

The Paleocene‐Eocene Thermal Maximum (PETM; ∼56 Ma) was a geologically short‐lived (∼150–250 kyr; e.g., Murphy et al., 2010; Röhl et al., 2007; Zeebe & Lourens, 2019) phase characterized by global warming, an enhanced hydrological cycle and biotic turnover (e.g., Carmichael et al., 2017; McInerney & Wing, 2011). A key feature of the event is a negative stable carbon isotope (δ^13^C) excursion (CIE) recovered in both marine (average: −2.8‰; bulk carbonate: −2.7‰; bulk marine organic matter: −4.1‰) and terrestrial records (average: −4.7‰; Koch et al., 1992; McInerney & Wing, 2011). In combination with dissolution of seafloor carbonates (e.g., Zachos et al., 2005), the CIE indicates the injection of large quantities of ^13^C‐depleted carbon (C) into the ocean‐atmosphere system (Dickens et al., 1995, 1997). Proposed sources for this carbon include methane hydrates (e.g., Dickens et al., 1995; Frieling et al., 2019; Lunt et al., 2011), terrestrial organic carbon (DeConto et al., 2012; Kurtz et al., 2003), thermogenic methane (Svensen et al., 2004), and volcanic CO_2_ (e.g., Bralower et al., 1997; Gutjahr et al., 2017) or a combination of sources (e.g., Panchuk et al., 2008; Sluijs et al., 2007). Many records show a rapid onset of the CIE followed by a “plateau” phase of stable δ^13^C values (e.g., Bowen et al., 2001; Thomas et al., 2002) with a duration of up to 170 kyr (Zeebe & Lourens, 2019; Figure 1). The plateau, or rather the lack of immediate recovery, implies a long‐term additional source of ^13^C‐depleted carbon, such as methane from hydrates, thermogenic sources or terrestrial carbon oxidation (Frieling et al., 2016; Lyons et al., 2019; Zeebe, 2013). The subsequent recovery to approximately background δ^13^C values may span up to ∼120 kyr (e.g., Farley & Eltgroth, 2003), though many records suggest stable postexcursion values within ∼100 kyr (Bowen, 2013).

The timescale of recovery (Bowen, 2013; Dickens et al., 1997; Zachos et al., 2005) as well as sedimentary evidence (e.g., Kelly et al., 2005; Ravizza et al., 2001) indicate that intensified weathering of terrestrial silicate rocks likely drove CO_2_ drawdown during the PETM (Zachos et al., 2005). However, the rapid recovery of the CIE observed in both marine and terrestrial records (e.g., Abdul‐Aziz et al., 2008; Giusberti et al., 2007), suggests that organic carbon (C_org_) burial played an important role as well, especially within the first 30–40 kyr of the recovery (Bowen, 2013; Bowen & Zachos, 2010). Along with terrestrial C_org_ burial (Bowen & Zachos, 2010), marine C_org_ burial might have been a major sink (e.g., John et al., 2008).

In the modern ocean, over 90% of all C_org_ burial in the marine realm takes place at depths shallower than 1,000 m (Sarmiento & Gruber, 2006). Climate change during the PETM likely led to increased weathering‐driven river inputs of phosphorus (P) to coastal zones (e.g., Carmichael et al., 2017; Khozyem et al., 2015; Sluijs et al., 2014), stimulating primary productivity. The resulting increase in organic matter remineralization near the seafloor, decreased bottom water oxygen concentrations, enhanced recycling of phosphorus from sediments through preferential release from organic matter and reduced mineral retention, and increased C_org_ burial (e.g., Ingall et al., 1993; Sluijs et al., 2008b, 2014). Warming, and stronger (salinity) stratification also contributed to a drop in seawater oxygen concentrations (e.g., Carmichael et al., 2017; Sluijs et al., 2006; Thomas et al., 2002). Given the high sea level across the late Paleocene and early Eocene (e.g., Miller et al., 2020), particularly during the PETM (e.g., Sluijs et al., 2008a), even more nutrients were likely trapped along margins due to their larger area and hence larger retention capacity (Tsandev & Slomp, 2009). Collectively, if marine C_org_ sequestration was of relevance to carbon drawdown during the PETM, it was most likely concentrated along continental margins.

Some studies suggest a rise in primary or export production (Bains et al., 2000; Ma et al., 2014) and mild deoxygenation (e.g., Chun et al., 2010; Pälike et al., 2014) in the open ocean, thus potentially increasing C_org_ sequestration. However, most observations point toward increased C_org_ burial on continental shelves and slopes (Dunkley Jones et al., 2018; John et al., 2008), notably in restricted basins such as the Arctic (Sluijs et al., 2008b) and in epicontinental seas such as the peri‐Tethys (Gavrilov et al., 1997). Sedimentary records from various margins point toward eutrophic conditions (e.g., Dickson et al., 2014; Gibbs et al., 2006; Nicolo et al., 2010; Schmitz et al., 1997a, 1997b; Soliman et al., 2011). Bottom waters at such locations were at least intermittently low in oxygen and in some cases even euxinic ([O_2_] = 0 and sulfidic), which in combination with elevated clay supply, enhanced C_org_ burial (Sluijs et al., 2014).

The rapid recovery of δ^13^C during the CIE through elevated C_org_ burial in marine sediments was recently assessed with two Earth System models. Using cGENIE, Gutjahr et al. (2017) and Dunkley Jones et al. (2018) assumed an ad hoc increase in burial to simulate the desired δ^13^C response. In an application of LOSCAR, Komar and Zeebe (2017) simulated a reduction in deep water O_2_ and associated increased P recycling and primary production as a mechanism for enhanced C_org_ burial. These studies conclude that the burial of ∼2,000 to 8,000 Pg (Pg) of C_org_ is required to reconstruct the initial, rapid δ^13^C recovery, in agreement with Bowen and Zachos (2010). However, neither cGENIE nor LOSCAR include a representation of the continental shelves and all C_org_ burial in these models occurs in deep marine sediments. As a result, the models do not capture the primary locale of C_org_ burial for the PETM which may affect both interpretations of the scale and nature of the driving mechanisms, as well as the actual magnitude of the C_org_ burial.

In this study, we first expand existing global scale data compilations of changes in primary production, and deoxygenation during the PETM (Carmichael et al., 2017; Dickson et al., 2012; Sluijs et al., 2014), based on the currently available published information and new data, with a focus on marginal sites. We then present a new biogeochemical box model with separate boxes for the open ocean and continental margins, the Arctic Ocean and the Eurasian Epicontinental Seas (EES). With the model, we simulate changes in primary production and water column redox conditions during the PETM, with a specific focus on the resulting burial of C_org_ on continental margins during the key first 40 kyr of the recovery phase.

## 2 Materials and Methods

### 2.1 Data Compilation

We generated new C_org_ records and seafloor oxygenation proxy records (C_org_/P_tot_, molybdenum, Fe/Al) for the PETM at seven sites (Figure S1 in Supporting Information S2), and combined them with published information on changes in primary production and redox conditions during the PETM (Table ST1 in Supporting Information S2). The compilation also includes new geochemical records for the deep North Atlantic (International Ocean Discovery Program (IODP) Site 1403) and marginal settings in the Pacific, Tethys, Atlantic, Arctic, Southern, and Indian Oceans (Lodo Gulch, Forada, Bass River, Lomonosov Ridge IODP Site M004, Ocean Drilling Program (ODP) Sites 1172 and 752; see Supporting Information S2 for site descriptions and methodology). For the recovery phase, we exclude sites lacking appropriate chronology.

To determine the redox conditions in the water column during the PETM, we use changes in sediment concentrations of redox‐sensitive trace metals (e.g., Mo, Fe/Al), changes in benthic foraminiferal assemblages and abundance, pyrite contents, sediment lamination, and the presence of biomarkers such as isorenieratene and derivatives. Increased values for the molar ratio of C_org_ over total P (C_org_/P_tot_) indicate increased preservation of C_org_ and reduced retention of P in sediments under low O_2_ conditions (e.g., Ingall et al., 1993). They are therefore used as a proxy for deoxygenation. The continued presence of benthic foraminifera is used as an indicator of bottom waters that were not permanently anoxic (e.g., Bernhard & Gupta, 1999). Laminations generally form as mesofauna and macrofauna disappear from sediments when bottom water oxygen is low, but not necessarily absent (e.g., Savrda & Bottjer, 1991). The presence of Mo and abundant pyrite in sediments generally indicates sulfidic sediments and anoxic bottom waters (Bertine, 1972; Crusius et al., 1996; Roychoudhury et al., 2003). Isorenieratene is a proxy for photic zone euxinia, though at shallow sites, photic zone euxinia likely extended to the seafloor (Sluijs et al., 2014). In this study, we attempt to narrow down redox classifications to no evidence for deoxygenation (oxic), reducing conditions without the complete absence of O_2_ (hypoxic) and evidence for no free O_2_ and/or sulfidic conditions (anoxic). Therefore, as an example, the presence of Mo at a site is interpreted as evidence for anoxia, and we forego the use of Mo concentrations for a further redox classification as done, for example, by Scott and Lyons (2012). For trace metals such as Fe/Al, an increase in values is interpreted as evidence for deoxygenation.

We exclude records that aim to reconstruct primary production using C_org_ records, as these can be influenced by changes in preservation of organic matter and sediment supply. We compile all studies that interpret proxy records purely as reflections of primary or export production, including those of barium concentrations and specific groups of plankton (e.g., foraminifera), to assess trophic levels, without a specific distinction between increased or high primary production.

### 2.2 Model Description

We developed an 11‐box biogeochemical model with representations of the coupled marine cycles of C_org_, P, and O_2_ to track changes in ocean biogeochemistry during the PETM in response to increased atmospheric CO_2_ and weathering. To this end, we formulated a steady state mass balance model for the late Paleocene and parameterized processes through simple rate laws (e.g., Ruvalcaba Baroni et al., 2014; Van Cappellen & Ingall, 1994). To simulate the PETM, we applied transient atmospheric pCO_2_ perturbations to assess the response to enhanced P weathering, higher temperatures, and increased stratification.

**Figure 2:**
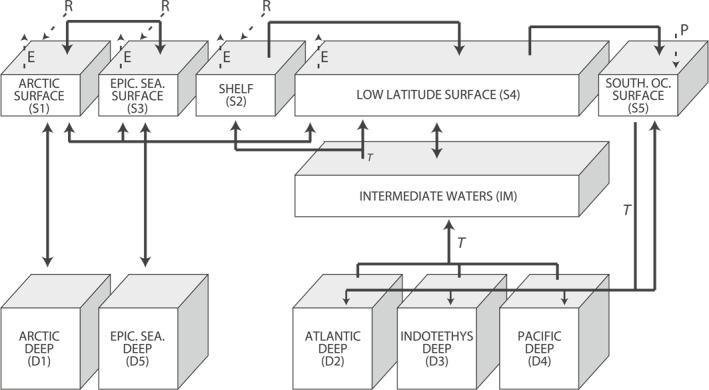
Setup of model boxes with names (codes) and fluxes determining the exchange between them. Fluxes between the boxes are indicated with solid lines and riverine fluxes (R), evaporation (E), and precipitation (P) are indicated by dashed lines. Thermohaline circulation assumes deep water formation in the Southern Ocean (Bice & Marotzke, 2001).

Our open ocean box layout is similar to that of the Walker and Kasting (1992) carbon‐cycle model with separate boxes for the surface waters in the midlatitude and low latitude ocean and Southern Ocean, a thermocline box and three boxes for the Atlantic, Indian, and Pacific deep oceans (Figure 2). The Indian Ocean box includes the Tethys Ocean and is referred to as Indotethys. The continental margins (shelf and slope), with the exception of the Arctic Ocean and EES (which includes the peri‐Tethys region), are represented by a single box. We hereafter refer to it as the continental margin. The Arctic Ocean and EES are each represented by two boxes, a shallow one that includes the shelf and slope area and a deep box. The continental margin, the surface Arctic Ocean, and the surface EES will be referred to collectively as the marginal boxes. This model setup allows for an evaluation of spatial variability in productivity changes and deoxygenation by modeling them separately for the shallow and deep, marginal, and open ocean and also for different ocean basins. Further details are provided in the Supporting Information S2.

### 2.3 Simulation Setup

**Figure 3:**
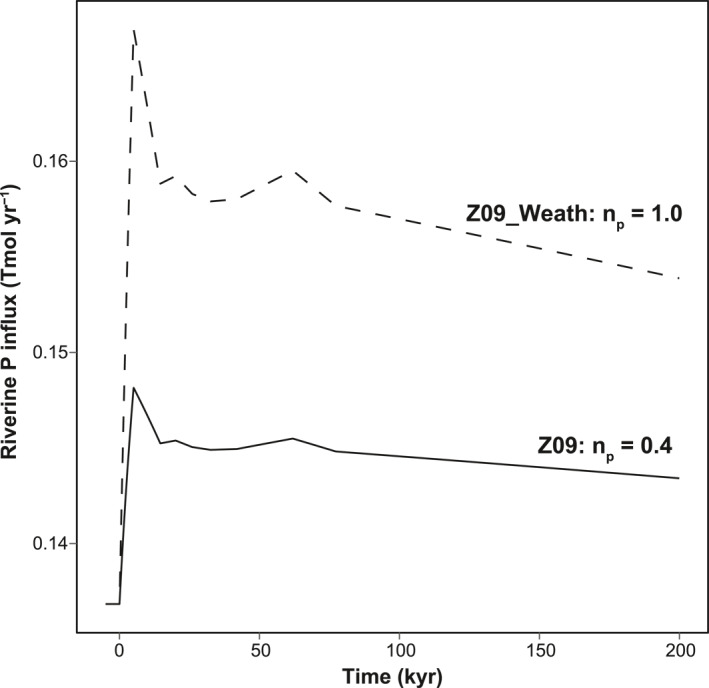
Overview of the increased riverine P influx that is used, together with rising temperatures and stratification, to simulate the The Paleocene‐Eocene Thermal Maximum (PETM) in our model. The standard scenario (Z09; solid line) uses a lower P weathering sensitivity of *n*_p_ = 0.4 than the Z09_Weath scenario in which *n*_p_ = 1.0.

The standard simulation which we present here is forced by the atmospheric CO_2_ curve as modeled by Zeebe et al., 2009 (Z09; Table ST2 in Supporting Information S2). As the model setup in this study does not include the complete exogenic carbon pool and therefore lacks a representation of greenhouse gas emissions, the values of atmospheric CO_2_ are prescribed for each time step. The increase in pCO_2_ during the PETM causes an increase in P_weath_ and, subsequently, total riverine P input, which closely follow the shape of the CO_2_ curve (Figure 3). Additionally, the accompanying increase in water temperature leads to a reduction in O_2_ solubility and we enforce an increase in stratification in the Arctic surface and EES surface boxes. The combined changes in P input, O_2_ solubility, and stratification cause the variations in primary productivity and deoxygenation associated with the PETM. The four key parameters of which the values chosen strongly affect our simulation results for the PETM are *f*_OrgP_ (0.75), *f*_CaP_ (0.4), *n*_p_ (0.4), and *f*_strat_ (S1: 0.1; S2: 1; S3: 0.4; Table ST2 in Supporting Information S2). The sensitivity analyses that we conducted before choosing these final values, which produce results that best fit the proxy records, are presented in Supporting Information S2. Additionally, we tested the impact of changes in these parameters in combination with riverine P inputs associated with three alternative atmospheric pCO_2_ curves (F16: Frieling et al., 2016; G17: Gutjahr et al., 2017; K170: which is the Z09 scenario but using the 170 kyr duration of Zeebe and Lourens (2019)) for the plateau of the CIE. Despite different emission scenarios, the CO_2_ curves produced by Frieling et al. (2016) and Gutjahr et al. (2017) are broadly similar to that of Zeebe et al. (2009). Similarly to Z09, the P input curves for the additional scenarios are similar in shape to the CO_2_ curves. The results of these simulations can also be found in Supporting Information S2.

We also performed five experiments using the Z09 scenario (Table ST2 in Supporting Information S2), with the aim to assess the sensitivity of C_org_ burial to of increased P supply, primary productivity, stratification, and reduced O_2_ solubility. In our first experiment (Z09_Weath), we enhanced the sensitivity of P weathering to atmospheric CO_2_, thereby increasing riverine P supply to the ocean. For this run, we increased the value of *n*_p_ from 0.4 to 1. In the second experiment (Z09_cOOPP), we kept open ocean (S4 and S5) primary productivity constant at the corresponding steady state value. As a result, the relative contribution of marginal (S1–3) productivity to total organic matter production increased. In this run, excess C_org_ burial is influenced solely by marginal productivity and deoxygenation in marginal and deep boxes. The third experiment tested the combined effect of decreased O_2_ solubility and increased P weathering, without increased stratification (Z09_cStrat). In this experiment, *f*_strat_ was set to 1 for all marginal boxes (S1–3), while *n*_p_ was kept at the standard value. For the next experiment (Z09_Weath_Only), we tested the impact of only enhanced P weathering on productivity and, subsequently, on C_org_ burial. To test this, we kept all boxes fully oxic (constant degree of anoxia, or DOA, and [O_2_] at steady state values), thus eliminating the contribution of preservation of C_org_ and P recycling. Our last experiment tested the effect of reduced O_2_ solubility under higher temperatures (Z09_O_2_Sol) assuming no increased stratification and no increased P weathering by setting *f*_strat_ to 1 for all marginal boxes (S1–3) and *n*_p_ to 0.

### 2.4 LOSCAR δ^13^C Simulations

To test the response of increased C_org_ burial rates on dissolved inorganic carbon δ^13^C, we used the carbon‐cycle box model LOSCAR (Zeebe et al., 2009). The C_org_ burial in the original LOSCAR version is constant throughout the simulations. For the purpose of this study, we changed this setup to include a time‐dependent C_org_ burial factor that emulated the relative changes in C_org_ burial, as simulated by our Z09 scenario. We tested a Z09 simulation with and without C_org_ burial, as well as two further emission scenarios, one for a larger second pulse of methane (see Komar & Zeebe, 2017) and one where the second pulse is caused by C_org_ oxidation (Lyons et al., 2019). Both of these scenarios are combined with Z09 C_org_ burial rates for the onset, plateau and first 40 kyr of the recovery phase. We use the LOSCAR δ^13^C fractionation value of −33‰ for C_org_ burial.

## 3 Results

### 3.1 Geochemical Data

The δ^13^C data for our newly generated PETM records show that we capture both the onset and recovery of the PETM at five out of seven sites (Figure S1 in Supporting Information S2). At the Lomonosov Ridge, molar C_org_/P_tot_ values well in excess of the Redfield ratio, and high Mo concentrations indicate severe deoxygenation. The high C_org_/P_tot_ values found at Lodo Gulch and the presence of Mo at Bass River and ODP Sites 1172 and 752 suggest some deoxygenation at these sites too. Average C_org_ contents are mostly at or below ∼0.5% at all sites except for Site 1172 and the Lomonosov Ridge where C_org_ contents range up to ∼1.3 and 3.4 wt %, respectively. For more details on the proxy records for our seven sites, see in Supporting Information S2.

### 3.2 Data Compilation

**Figure 4:**
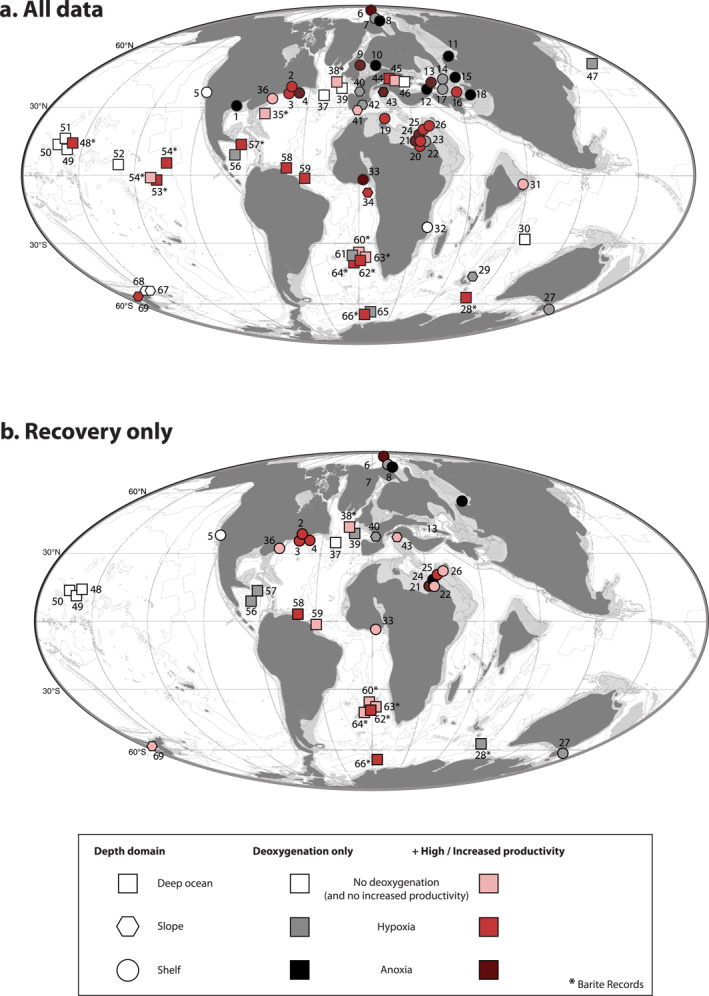
Global overview of sites of proxy records on changes in primary productivity (PP) and bottom water redox conditions for the PETM. Records for all phases of the PETM (a) and records for the recovery as identified from the δ^13^C excursion (b) are indicated separately. The lightest colors indicate oxic conditions or sites where no data on deoxygenation is available. Intermediate shades are used for hypoxic sites while the darkest shades indicate proxy evidence for anoxic or euxinic conditions (see text for criteria). Gray colors are used for sites without data on PP changes and red colors indicate proxy evidence for high or increased PP. Sites for which there is no proxy evidence for deoxygenation or increased productivity are left white. Sites with records that use barite to reconstruct PP are indicated by stars (*) and sites where proxy records offer contradicting results by question marks (?). Symbol shapes represent the depth domain: shelf (circle), slope (polygon), and deep (square). For the full reference list and site names (here indicated by numbers) see Table ST1 in Supporting Information S2. Separate maps on deoxygenation and productivity changes can be found in Figure S2 in Supporting Information S2. Map after Markwick (2007), modified by Sluijs et al. (2014).

Most sites in in our compilation (Figure 4a) experienced some degree of deoxygenation during the PETM. Hypoxic conditions on the seafloor at open ocean sites are concentrated mainly in the areas of the Equatorial Pacific and Atlantic, Walvis Ridge in the Southern Atlantic and the Southern Ocean. Most sites on the continental shelf and slope experienced hypoxia at some stage during the PETM, with only a few (<5) exceptions. Intermittent to permanent anoxia developed at sites in the Arctic Ocean, the North Sea, the Peri‐Tethys, the New Jersey shelf, and the North African shelf. However, sites on the New Jersey and North African shelves provide evidence for hypoxia rather than anoxia. Lower photic zone euxinia was identified at a total of four sites.

Signs of eutrophication are found in all basins and at all water depth intervals, during different stages of the PETM. About 13 sites experienced high productivity throughout the entire PETM (Table ST1 in Supporting Information S2). Clusters of high productivity occur around the Walvis Ridge, on the New Jersey Shelf, and the North African Shelf. Most sites with evidence for increased productivity also experienced deoxygenation, with a few exceptions. A much smaller number of records capture the recovery phase of the PETM (Figure 4b). At some sites, notably in the deep ocean and the (peri)Tethyan realms, proxies indicate oxic conditions during the recovery, but a large number of sites in various ocean basins, notably on the North African Shelf, the New Jersey Shelf, and in the Arctic remained hypoxic or anoxic.

**Figure 5:**
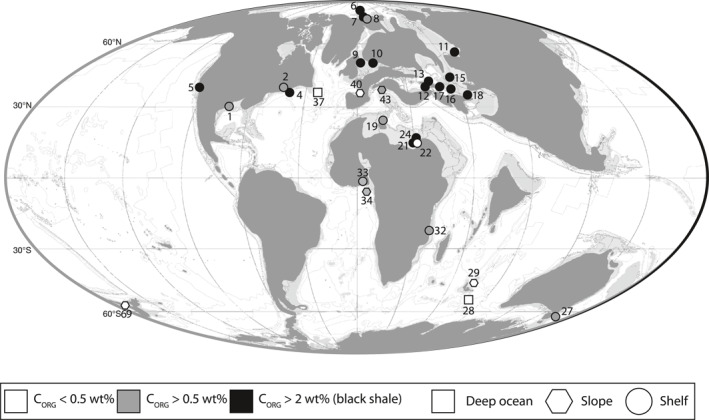
Compilation of maximum sediment C_org_ contents for the PETM. Colors represent maximum values for each site: less than 0.5 wt % (white), more than 0.5 wt % (gray), and more than 2 wt % (black). Shapes indicate the depth domain: shelf (circle), slope (polygon), and deep (square). For the full reference list and site names (here indicated by numbers) see Table ST1 in Supporting Information S2. Map after Markwick (2007), modified by Sluijs et al. (2014).

Overall, there is abundant evidence for eutrophication and deoxygenation in marginal and restricted environments, also during the recovery phase of the PETM. This contributed to high sediment C_org_ contents in these environments (Figure 5). Records containing *C*_org_ > 0.5 wt %, and especially *C*_org_ > 2 wt %, are found mainly in marginal environments during the PETM (Figure 5).

### 3.3 Modeling Results

**Figure 6:**
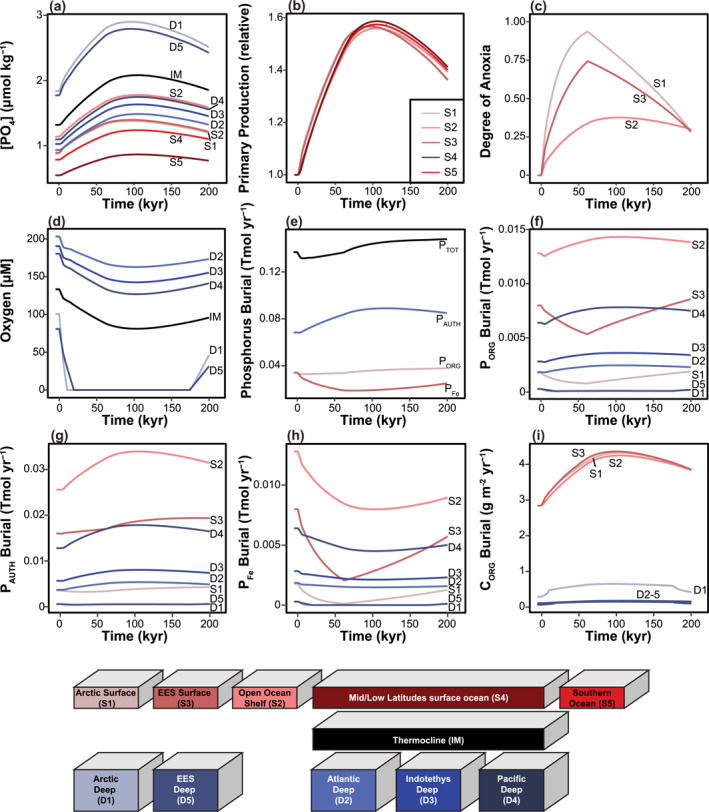
Key results for PETM simulation Z09: phosphate concentrations in μmol kg^−1^ (a), relative change in primary productivity (b), degree of anoxia for the marginal boxes (c), oxygen concentrations in μM for the deep boxes (d), total phosphate burial rates for the different pools in Tmol yr^−1^ (e), organic P (P_org_) burial rates in Tmol yr^−1^ (f), authigenic P (P_auth_) burial rates in Tmol yr^−1^ (g), iron‐bound P (P_Fe_) burial rates in Tmol yr^−1^ (h), and organic carbon (C_org_) burial rates in g C m^−2^ yr^−1^ (i). Red colors are used for surface boxes, black for the thermocline and blue colors for the deep boxes. The color association is also shown in boxes at the bottom of this figure.

We present results for the Z09 simulations here (Figure 6). Those for the F16, G17, and K170 simulations are very similar and can be found in Supporting Information S2.

#### 3.3.1 Z09 Simulation

Dissolved PO_4_ concentrations in all basins and primary production rates in the surface boxes increase by a factor of ∼1.6 during the first 80–100 kyr of the event, before decreasing (Figures 6a and 6b). Phosphate concentrations become highest in the deep boxes of the restricted basins (D1, D5; ∼3 μmol kg^−1^), somewhat lower in the thermocline (>2 μmol kg^−1^) and remain low in the Southern Ocean (<1 μmol kg^−1^; Figure 6a). Total primary productivity increases from ∼46 to ∼75 Pg C yr^−1^. This increase results in excess production of ∼4 × 10^6^ Pg C, with 79% produced in S4 and 11% in S2.

Deoxygenation occurs in all surface (Figure 6c) and deep (Figure 6d) boxes. The DOA is highest in box S1 (0.94), compared to S2 (0.38) and S3 (0.74). The DOA for S2 begins to decrease at about 100 kyr. For S1 and S3, the recovery begins around 60 kyr into the event. Oxygen concentrations in the deep Arctic Ocean (D1) and EES (D5) decrease to zero within the first ∼10 and ∼20 kyr of the PETM simulation, respectively. Both boxes remain fully anoxic until ca. 30 kyr before the end of the event. The thermocline becomes nearly hypoxic (min. 84 μmol kg^−1^). Oxygen concentrations in the deep open ocean boxes decrease but remain >60 μmol kg^−1^. Oxygen concentrations for the thermocline and deep open ocean (IM, D2–4) begin their recovery from ∼100 kyr onward.

The burial of P_tot_ decreases at the onset of the PETM and remains below the late Paleocene level for the first ∼60 kyr of the event, followed by a rise (Figure 6e). Variations in total P_org_ burial are minor compared to the rise in P_auth_ and decrease in P_Fe_ burial. Interestingly, while P_org_ burial increases in the deep ocean boxes D2, D3 and D4, and the shallow box S2, it decreases in D1, S1, and S3 (Figure 6f). The burial of P_auth_ shows little change in D1, D5, and S1 but increases in all the other boxes (Figure 6g). Burial of P_Fe_ decreases in all boxes, but most prominently in S2 and S3 (Figure 6h).

The rate of C_org_ accumulation increases in all boxes (Figure 6i). In the marginal boxes, the rate increases from an average of 2.9 to 4.3 g C m^−2^ yr^−1^. In the deep boxes, the largest change occurs in the restricted basins, where rates more than double from 0.29 to 0.65 g C m^−2^ yr^−1^ in D1 and from 0.07 to 0.15 g C m^−2^ yr^−1^ in D5.

**Figure 7:**
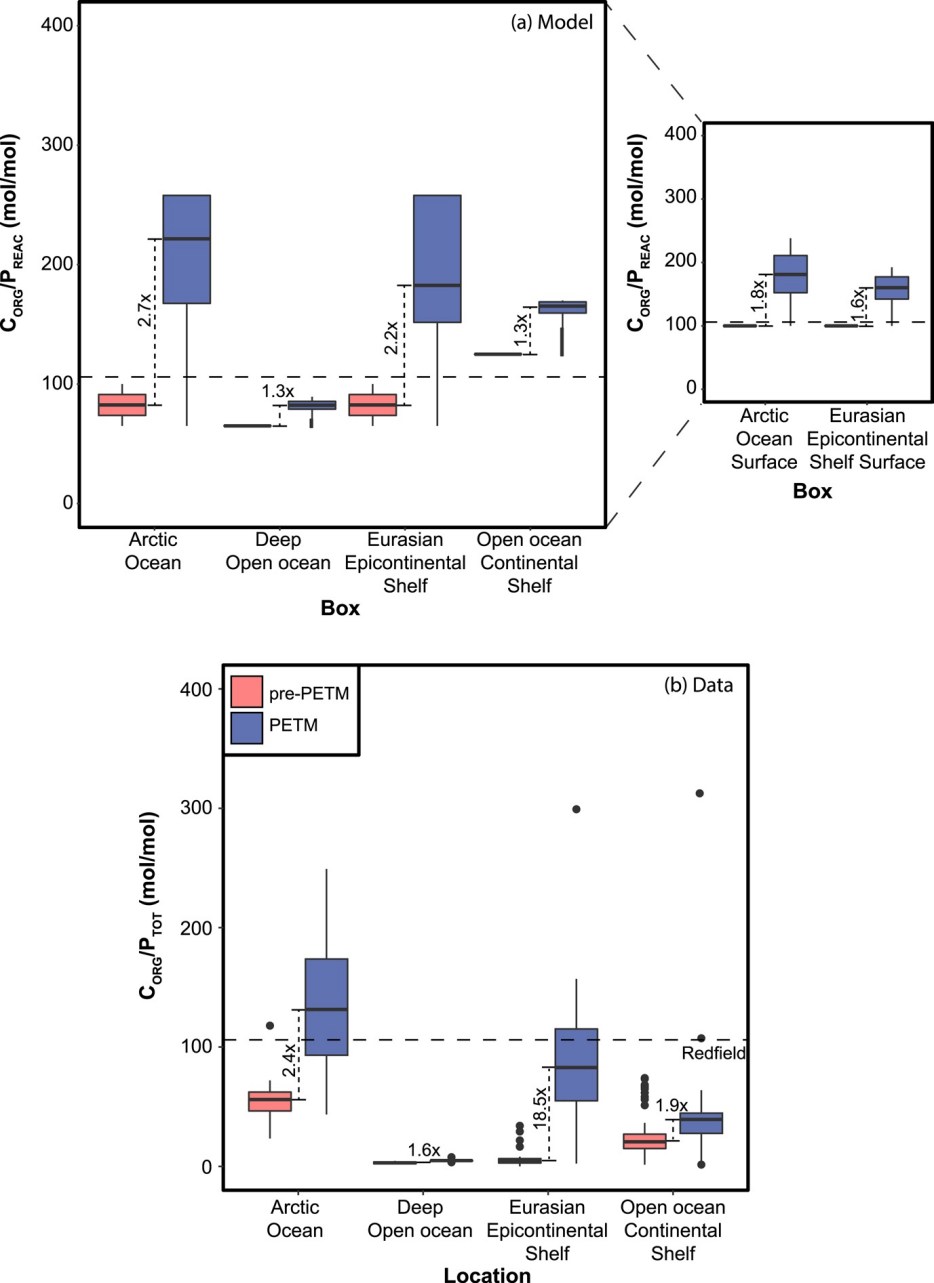
Simulated and measured molar (mol/mol) values of C_org_/P_tot_ for marginal and deep ocean regions based on our model scenario Z09 (a) and data from this study and Dickson et al. (2014) (b). In the data set, total P (P_tot_) is equal to reactive P (P_reac_) plus detrital P. The latter phase is not included in the total P in our model. Values are shown for the late Paleocene (pink) and the PETM (blue). The dashed line indicates the Redfield value (106). The relative change in median values is indicated. The inset for panel (a) shows the values for the Arctic and EES surface boxes, which in (a) are combined with the values for the respective deep boxes. Horizontal lines within the boxplots indicate median values.

Burial C_org_/P_tot_ values during the PETM generally exceed the Redfield ratio of 106 in all boxes, except for the deep open ocean (D2–4; Figure 7a). Maximum values (260 mol/mol) are reached in the Arctic and Eurasian Epicontinental Seaway and the maximum relative increase (2.4) occurs in the Arctic. The lowest maximum value (83 mol/mol) and the lowest relative change (1.27) occur in the deep open ocean.

#### 3.3.2 Excess C_org_ Burial

**Figure 8:**
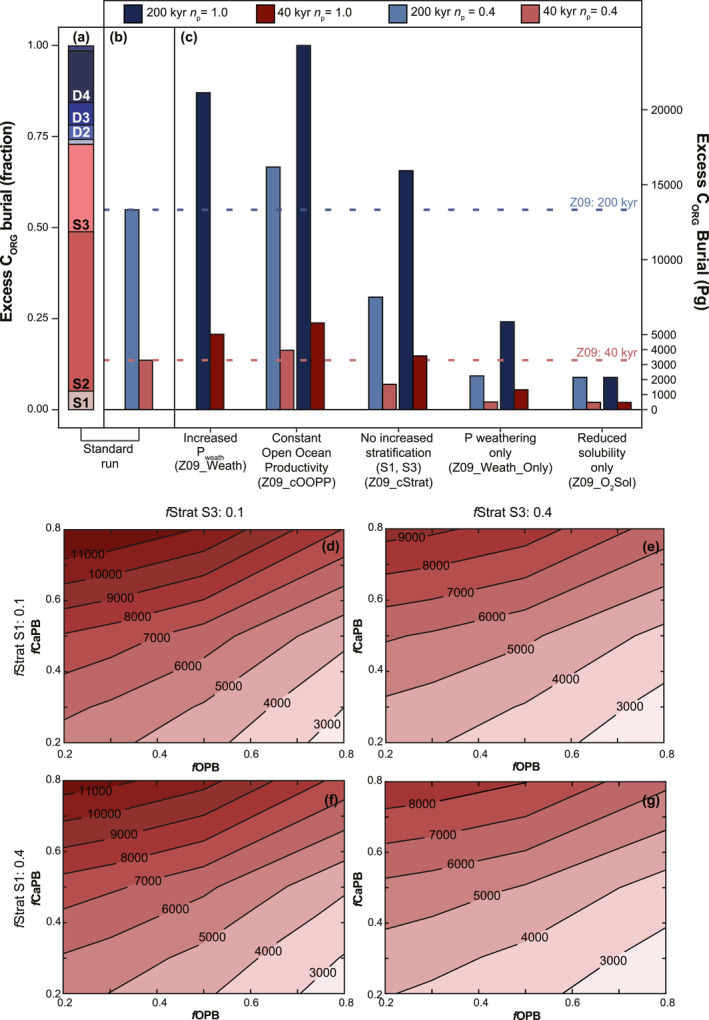
Barplot for excess C_org_ burial associated with the PETM for the standard simulation Z09 (a, b) and the sensitivity analyses (c) in units of Pg C and as a relative fraction when compared to Z09. The response of C_org_ burial for the first 40 kyr of the recovery to different degrees of stratification in S1 and S3, as well as varying redox sensitivity for P burial is shown in (d–g). The codes for the boxes contributing to the excess C_org_ burial are as described in Figure 2. Blue colors are used for excess burial across the entire PETM while red indicates excess burial during the first 40 kyr of the recovery. Light colors in (b, c) are used for the lower P weathering sensitivity (*n*_p_ = 0.4) while darker shades correspond to the higher sensitivity (*n*_p_ = 1.0). See the Methods section for an explanation on the calculation of excess burial. Scenario codes are given in brackets and explained in Table ST2 in Supporting Information S2.

Most excess burial of C_org_ for the standard scenario (Z09) occurs in the marginal boxes (Figure 8a), which account for more than 70% of total burial. Over the entire PETM, excess burial amounts to ∼13,300 Pg C (Figure 8b). During the first 40 kyr of the recovery, excess burial is 3,300 Pg C. For the sensitivity tests, we show results for simulations with a P weathering sensitivity of either *n*_p_ = 0.4 (as in the standard run) and, only where it is explicitly stated, *n*_p_ = 1.0 (as in Z09_Weath; Figure 8c). An increase in weathering sensitivity from 0.4 to 1.0 generally increases the amount of excess C_org_ burial. When this increase is applied to the standard scenario (Z09_Weath), it results in total excess burial of ∼21,100 Pg C, while for the first 40 kyr of the recovery excess burial increases to 5,000 Pg C. Generally, excess burial is at least 1.4 times higher for a weathering sensitivity that is 2.5 times higher. When open ocean productivity is kept constant (Z09_cOOPP), excess C_org_ burial shifts toward the restricted basins. Due to a decreased retention of phosphorus in the open ocean, more of it becomes available for production in the restricted basins instead. Total excess C_org_ burial is ∼16,000 Pg C for the entire PETM and ∼4,000 Pg C for the early recovery.

The effect of changes in stratification on excess C_org_ burial is large and is required to reproduce the spatial extent and severity of deoxygenation in the Arctic and EES. Without increased stratification in S1 and S3 (Z09_cStrat), total excess C_org_ burial over the entire PETM is ∼7,500 Pg C and ∼1,700 Pg C of this is buried during the 40 kyr interval. The CO_2_‐driven increase in P_weath_ and associated riverine input of P is the only factor controlling biogeochemical changes in the ocean in the model run with oxic conditions and without increased stratification (Z09_Weath_Only). Total primary productivity increases to ∼53 Pg C yr^−1^ and excess C_org_ burial amounts to ∼2,250 Pg C (entire event) and ∼500 Pg C (first 40 kyr of recovery). The effect of reduced O_2_ solubility on C_org_ burial, due to warming only (Z09_O_2_Sol), is similar to that of increased P_weath_ only (Z09_Weath_Only). Excess C_org_ burial by the end of the PETM is ∼2,100 Pg C and roughly ∼480 Pg C are buried during the 40 kyr early recovery interval.

The sensitivity tests show that increased weathering of P is a key driver of biogeochemical change during the PETM. However, the redox‐driven recycling of P amplifies this effect, resulting in the patterns of increased productivity and deoxygenation that we observe in the data. The degree of redox sensitivity for P burial, as well as the degree of deoxygenation in key areas of burial further affect the amount of C_org_ burial (Figures 8d–8g and Figure S4 in Supporting Information S1).

### 3.4 LOSCAR δ^13^C Simulations

**Figure 9:**
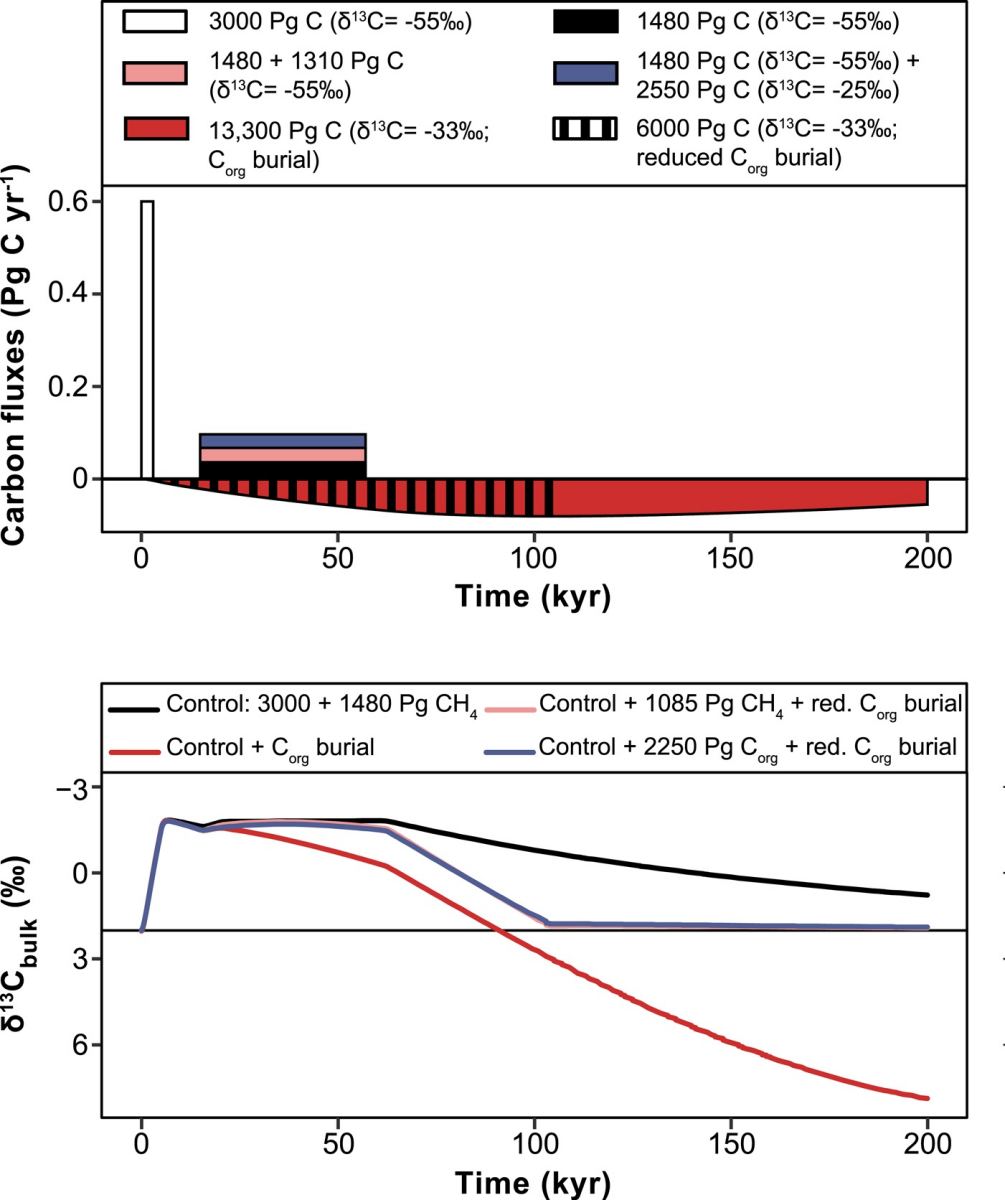
Bulk carbon isotope response to four PETM forcing scenarios. The forcing scenarios (a) include methane (CH_4_; δ^13^C = −55‰) and C_org_ (δ^13^C = −25‰) emissions, and C_org_ burial. White bar: an initial pulse of 3,000 Pg C of CH_4_ emission (Zeebe et al., 2009), used in all scenarios. Black bar: a “leak” of 1,480 Pg C of CH_4_ (Zeebe et al., 2009). Pink bar: an additional “leak” of 2,020 Pg C of methane. Blue bar: an additional “leak” of 1,020 Pg C of CH_4_ and 2,400 Pg C of C_org_. Purple bar: reduced burial, excess C_org_ burial of 6,000 Pg C, over the onset, plateau and first 40 kyr of the recovery. Red bar: excess C_org_ burial of 7,300 Pg C, together with the purple bar it accounts for the burial of 13,300 Pg C over the entire PETM. The bulk δ^13^C responses (b) correspond to combinations of these scenarios. Black line: Control scenario with a forcing of 3,000 Pg C plus 1,480 Pg C of CH_4_. Red line: Control scenario with the inclusion of the complete C_org_ burial scenario simulated in this study. Pink line: Control scenario plus 2,020 Pg C of CH_4_ and reduced burial. Blue line: Control scenario plus 1,020 Pg C of CH_4_ and 2,400 Pg C of C_org_, with reduced burial. 0 kyr: Onset.

The inclusion in LOSCAR of C_org_ burial rates simulated in this study, results in a preferential removal of ^13^C‐depleted carbon and thus an earlier and more rapid recovery of the CIE (red line in Figure 9). The shape of the CIE broadly matches observations (Figure 1). However, by the end of the PETM, δ^13^C values have increased by 2‰ above the preevent value, which is in stark contrast with observations and thus implies the need for the addition of several thousand Pg of ^13^C‐depleted C during the plateau and recovery phases to match the shape of the CIE. Truncating C_org_ burial rates after the first 40 kyr of the recovery (blue, pink lines), leads to a better match with the target. An addition of ∼1,000 Pg C with a δ^13^C value of −55‰ (blue line), or 2,400 Pg C of −25‰ (pink line), results in a plateau phase similar to the scenario without C_org_ burial.

## 4 Discussion

### 4.1 Primary Production

Our data compilation shows that primary production increased in both open ocean and marginal marine areas during the PETM (Figure 4). Roughly 70% of all studied sediment records reveal increased production based on at least one proxy. Some work has suggested that barite records (partly) record export rather than primary production (Ma et al., 2014)—though it seems unlikely that export production increased without a pronounced increase in primary production, particularly in a warmer water column (e.g., Laws et al., 2000)—or a global increase in the Ba inventory (Dickens et al., 2003; Frieling et al., 2019). Even when barite records are excluded, 60% of all sites still show an increase in primary production. Importantly, almost two thirds of all high or increased productivity sites are located on the continental margin and in the restricted basins of the EES and Arctic Ocean, where almost all sites show an increase during the PETM. This is especially true for areas such as the North African shelf (sites 19–26) and the New Jersey shelf (sites 2–4), where increased productivity marks all sites but one (23).

Our simulations indicate an equal relative increase of primary productivity in all surface boxes. The absolute increase in maximum rates of primary production per unit of surface area is highest in marginal boxes (S1–3), but most production takes place in the low and mid latitudes of the open ocean (S4; Table ST3 in Supporting Information S2). These environments cover the largest surface area of the ocean and proxy records for high or increased primary production are found in the North and the South Atlantic, as well as the tropical Pacific.

### 4.2 Deoxygenation

Deoxygenation is recorded by proxies at 90% of the sites compiled for this study (Figure 4). Sulfur isotope ratios have been interpreted to suggest that large parts of the ocean became sulfidic during the PETM (Yao et al., 2018) with an expansion of the OMZ to 10–20% of the ocean volume (modern: 1%). While there is local evidence for hypoxia at intermediate depths in the open ocean (Figure 4) there are no signs of euxinic, or even anoxic conditions on such a scale. (Intermittent) euxinic conditions are almost entirely restricted to specific sections of the continental shelf: the Arctic, the eastern EES and peri‐Tethys region, the North Sea, the North African Shelf, and the Gulf of Mexico.

The results of our simulations are in general agreement with the data, as all boxes show signs of deoxygenation. The deep boxes of the open ocean also exhibit a decrease in [O_2_] but remain oxic (>60 μM) during the PETM. Intermediate waters (box IM) also remain oxic. As our model provides average changes for all basins, this result is not inconsistent with the observed hypoxic areas ([O_2_] < 60 μM) as others remained oxic (e.g., Pälike et al., 2014). The deep Arctic and EES become fully anoxic and the DOA rises for all surface boxes, consistent with observations. At present, there is no data available for the deep Arctic basin, however our model does not allow for an oxic, or even hypoxic, deep Arctic when the surface box experiences a large increase in DOA. As surface deoxygenation for the Arctic is supported by work on multiple sites (Figure 4; see Table ST1 in Supporting Information S2 for all references), it is likely that the deep Arctic experienced similar conditions.

The changes in the hydrological cycle that are associated with the PETM likely resulted in increased river runoff to the marine realm which, in combination with higher temperatures, would have caused water column stratification (e.g., Sluijs et al., 2006). In our model, stratification plays a large role in increasing the DOA of surface boxes (Table ST3 in Supporting Information S2). Marginal environments where evidence suggests that salinity varied, are mainly located in the Arctic (Harding et al., 2011; Pagani et al., 2006; Sluijs et al., 2008b) and on the New Jersey Shelf (Kopp et al., 2009; Sluijs & Brinkhuis, 2009). But evidence of intense hydrological change on land (e.g., Bowen et al., 2004; Chen et al., 2018; Foreman et al., 2012; Schmitz & Pujalte, 2007) and an increase in the supply of terrestrial siliciclastic and organic material to the margins has been found in numerous locations (see overview in Sluijs et al. (2014)), likely accompanied by large‐scale (seasonal) salinity stratification along margins. Very strong stratification is required in our simulations to create severe anoxia in the Arctic, so as to correspond to the conditions that were dominant at Lomonosov Ridge (Dickson et al., 2012; Sluijs et al., 2006, 2008a, 2008b; Stein et al., 2006) and Spitsbergen (Cui et al., 2011; Harding et al., 2011). A somewhat lower DOA for the EES, again mostly due to stratification, encompasses the larger range of redox conditions deduced from proxies for this region (Figure 4), though anoxia occurred (intermittently) at nearly half of all sites there. Without stratification, our model does not capture such conditions for the EES. Despite the fact that we did not enforce stratification on the continental margin (box S2), stratification elsewhere also results in a somewhat higher DOA for S2.

### 4.3 Phosphorus Recycling

Phosphorus is considered the main limiting nutrient in the ocean on long timescales (Tyrrell, 1999). Records of C_org_/P_tot_ indicate that P recycling relative to C_org_ increased from the late Paleocene into the PETM (Figure 7b). Typical values of C_org_/P_tot_ were lower than those in modern anoxic to euxinic basins, however, where they generally far exceed the Redfield ratio, reaching values of up to 400 mol/mol (Algeo & Ingall, 2007). Values in excess of the Redfield ratio (generally < 300 mol/mol) occur at Guru Fatima, Kheu River (Dickson et al., 2014), and the Arctic and Lodo Gulch (Figure S1 in Supporting Information S2). This confirms that throughout the PETM, complete anoxia was only experienced locally, while most locations experienced at most a switch to hypoxia.

Overall, the absolute range of C_org_/P_tot_ simulated by our model corresponds well to the range of the data (Figure 7), when taking into account that we do not model detrital P (Supporting Information S2). In contrast, the relative increase in the modeled median C_org_/P_tot_ value is much lower than the increase in the data. This is especially true for the EES where the relative change in median data values is more than a factor 18, versus a modeled increase of 1.6–2.5 times the late Paleocene median C_org_/P_tot_ value (Figure 7b; Dickson et al., 2014). Here, we must add that in addition to deoxygenation and P recycling, an increase in the input of detrital P would lower sedimentary C_org_/P_tot_, while increased terrestrial organic matter fluxes would have the opposite effect (Ruttenberg & Goni, 1997; Burdige, 2005). An increase in the contribution of terrestrial organic material is found at some sites during the PETM (e.g., Arreguín‐Rodríguez et al., 2014; Crouch et al., 2003). Furthermore, the locally intensified hydrological cycle (Carmichael et al., 2017) would have led to an increased influx of both detrital P and terrestrial organic matter, with uncertain effects on marine C_org_/P_tot_. The extremely large change for the EES in particular, combined with an increased abundance of terrestrial biomarkers and highly weathered lithogenic material (Dickson et al., 2014), suggests that PETM values were affected by an increase in terrestrial material. We therefore infer that the strength of P recycling within our model falls within a reasonable range for the PETM.

The ability of biota to utilize the excess availability of P during the PETM may have depended on the cycling of other nutrients such as N and Fe as well. Nitrogen isotope (δ^15^N) records suggest an increased availability of ammonium in the photic zone and potentially a P‐driven increase in N_2_ fixation during the PETM (Junium et al., 2018; Knies et al., 2008). The cycling of Fe in the past ocean is even harder to trace, however local transitions to a (seasonally) drier climate (Carmichael et al., 2017) likely resulted in enhanced wind‐driven Fe deposition in nearby marine environments. Additionally, deoxygenation may have led to elevated fluxes of bioavailable Fe from continental shelf sediments to ocean waters (Homoky et al., 2012; Lenstra et al., 2019; Raiswell et al., 2018).

### 4.4 Organic Carbon Burial

In our standard scenario, which satisfactorily reproduces the trends and patterns of primary productivity, deoxygenation, and P recycling seen in data, the combination of these processes results in a total burial of 45,300 Pg C (Table ST3 in Supporting Information S1) and excess burial of ∼13,000 Pg C across the entire PETM (Figure 8b). The fraction of produced C_org_ that is buried increases from 1% (late Paleocene) to 3% (Table ST3 in Supporting Information S2). An increase in riverine P input from ∼0.15 Tmol P yr^−1^ (Z09) to ∼0.17 Tmol P yr^−1^ (Z09_Weath), leads to a rise in excess C_org_ burial of 7,800 Pg C across the entire PETM. Our results emphasize the importance of redox‐driven P recycling for the burial of C_org_. When P burial is decoupled from [O_2_] and DOA (Z09_Weath_Only), increased terrestrial nutrient input and subsequently higher primary productivity, fuel excess C_org_ burial that is ∼16% of the Z09 value (Figures 8b and 8c). The simulated values for C_org_ burial also depend on the chosen values for model parameters on stratification and redox sensitivity of P burial (Figures 8d–8g; Supporting Information S2). Changes in these parameter values, however, shift the trends and patterns of, for example, deoxygenation (Figures S4b and S4c in Supporting Information S2), reducing the correspondence with proxy data.

More than 70% of all excess burial in our model occurs in the three marginal boxes (S1–3; Figure 8a). Despite its large area and consequently large production, burial in the Pacific Ocean makes up only 10% of excess C_org_ burial. In fact, when open ocean production is kept constant (Z09_cOOPP), the resulting increase in nutrients in the marginal boxes leads to increased total global production and excess C_org_ burial. Such a change in the trophic resource continuum was postulated by Gibbs et al. (2006) to explain the different productivity trends between Wilson Lake on the New Jersey Shelf and ODP Site 1209 in the Pacific Ocean. In contrast to previous suggestions (e.g., Bains et al., 2000), our study further supports the notion that deep ocean organic carbon burial played a very minor role in the recovery of the PETM.

Our simulated total C_org_ burial for the Z09 scenario is at least 1.7 times higher than the burial estimated from our data compilation (Supporting Information S2). The amount of total excess C_org_ burial in our Z09 simulation (13,300 Pg C) is higher than that determined from marginal marine sediment records and previous model simulations (4,000–6,000 Pg C; John et al., 2008; Komar & Zeebe, 2017). By contrast, excess C_org_ burial in the Arctic (700 Pg C in surface; 870 Pg C in total) is very similar to the value (770 Pg C) of Sluijs et al. (2008b). We must note here that the sediment cores cover just a minor fraction of the full extent of the margins and their mass accumulation rate values may not be representative for other localities. Additionally, if we calculate the mass accumulation rates for our model, the maximum change in rate between the late Paleocene and the PETM are similar between our model and the data: 1.51 g m^−2^ yr^−1^ (Sluijs et al., 2008b) and 1.49 g m^−2^ yr^−1^ for the Arctic (Figure 6i), and 1.1 g m^−2^ (John et al., 2008) and 1.4 g m^−2^ for the continental margin (Figure 6i). As it is this difference that determines excess burial, and our rates fall well within the modern ranges (e.g., Alperin et al., 2002; de Madron et al., 1999), we are confident that our C_org_ burial results are realistic for the PETM. Collectively, we conclude that our simulated C_org_ burial, caused by changes in productivity and deoxygenation that are in good agreement with field data, is realistic for the PETM.

### 4.5 Can C_org_ Burial Explain the Shape of the CIE?

The burial of C_org_, and the δ^13^C signature that is used, determines the reconstruction of the CIE and the estimation of carbon emissions. Zeebe et al. (2009) proposed a methane addition scenario of 4,480 Pg C, which Komar and Zeebe (2017) adjust to 5,500 Pg C following the inclusion of C_org_ sequestration (δ^13^C_org_: −33‰, as in this study). For their work on C_org_ burial and its effect on δ^13^C, Gutjahr et al. (2017) used a δ^13^C_org_ value of −30.5‰, resulting in an, mostly volcanic, emission scenario of 10,000 Pg of C_org_, whereas Dunkley Jones et al. (2018) used a δ^13^C_org_ −22‰. In this study, the burial of 13,300 Pg C, in excess of the late Paleocene, combined with the Zeebe et al. (2009) methane emission scenario, captures the general CIE shape and the rapid recovery but not the stable CIE plateau (Figure 9).

Previous work (Bowen & Zachos, 2010; Dunkley Jones et al., 2018; Gutjahr et al., 2017; Komar & Zeebe, 2017) has highlighted that the burial of C_org_ (at least 2,000 Pg C but up to 8,000 Pg) is required to explain the relatively fast recovery within the first 30–40 kyr of the CIE. Our main PETM simulation, that now includes the correct locus for marine C_org_ burial, shows that changes in productivity, deoxygenation and P recycling, as evident in proxy data, could have supported such an amount of excess burial (3,300 Pg C; total burial 9,600 Pg C; Table ST3 in Supporting Information S2) within 40 kyr, buried mainly on the continental margin and the EES. The range of values for our sensitivity analyses is ∼500–6,000 Pg C, most of which is caused by redox feedbacks on C_org_ and P burial (Figure 8).

The total increase of δ^13^C during the recovery interval results in an overshoot relative to pre‐PETM values. This occurs largely in the second half of the recovery, a time interval for which there are few constraints on the extent and degree of productivity and deoxygenation (see Table ST1 in Supporting Information S2). When C_org_ burial rates up to and covering the first 40 kyr of the recovery are used, this δ^13^C overshoot is not simulated (Figures 1 and 9). This truncation of C_org_ burial rates also results in lower total C_org_ burial (22,100 Pg C), which is close to our maximum burial estimates from the data ( in Supporting Information S2), and lower total excess C_org_ burial (∼6,000 Pg C), similar to the 5,000 Pg C suggested by Komar and Zeebe (2017). We therefore propose that the higher burial estimate of 13,300 Pg C is an overestimation caused by a lack of appropriate data. Importantly, the excess C_org_ burial during the key 40 kyr phase reproduces the more rapid recovery of δ^13^C of ∼2‰ as noted by Bowen and Zachos (2010). Using methane (δ^13^C: −55‰) or methane in combination with C_org_ (δ^13^C: −25‰) as sources of C emissions, and taking into account a total excess burial of 6,000 Pg, our simulations require 5,790 or 7,030 Pg C to reproduce the CIE, respectively. A mass of at least 10,000 Pg C in volcanic emissions (δ^13^C: −11‰) is needed to reproduce the CIE in combination with increased C_org_ burial (Gutjahr et al., 2017).

## 5 Conclusions

We compiled new and published proxy data for eutrophication and deoxygenation during the PETM and combined the results with biogeochemical modeling to simulate the effect on phosphorus and carbon burial over the event. We find that signs of increased primary productivity and spreading low oxygen conditions are largely concentrated in marginal and restricted sections of the ocean following the onset of the PETM and its recovery (final ∼120 kyr). Our modeling results demonstrate that this spread of productive, low oxygen waters on the continental margin, the Arctic Ocean and Eurasian Epicontinental Seas could be caused by increased CO_2_‐driven riverine input of phosphate and water column stratification, further enhanced by phosphorus recycling linked to deoxygenation. Data and simulations show that deep sea organic carbon burial was quantitatively unimportant during the PETM. Our best estimate for excess C_org_ burial across the PETM is 6,000 Pg C. Finally, our model suggests that eutrophication and deoxygenation within the first 40 kyr of the recovery phase could have led to excess sequestration of 3,300 Pg of C_org_, which corroborates previous studies in showing that C_org_ burial of this order of magnitude is required to explain a rapid increase in global exogenic δ^13^C at the beginning of the recovery phase. Further substantiation of this burial mass would require additional C_org_ records, especially from the Southern Hemisphere and the Arctic, and detailed age models for marginal sites to allow insight into changes in accumulation rates during the recovery phase of the PETM.

## Supporting information

Supporting Information S1

Table S1

Table S2

Table S3

Table S4

Table S5

## Data Availability

All new data are available online at PANGAEA (Major element composition: https://doi.org/10.1594/PANGAEA.929261, https://doi.org/10.1594/PANGAEA.929308, https://doi.org/10.1594/PANGAEA.929255, https://doi.org/10.1594/PANGAEA.929263, https://doi.org/10.1594/PANGAEA.929303, https://doi.org/10.1594/PANGAEA.929305, https://doi.org/10.1594/PANGAEA.929259; organic carbon and carbonate: https://doi.org/10.1594/PANGAEA.929262, https://doi.org/10.1594/PANGAEA.929258, https://doi.org/10.1594/PANGAEA.929264, https://doi.org/10.1594/PANGAEA.929304, https://doi.org/10.1594/PANGAEA.929307, https://doi.org/10.1594/PANGAEA.929260). Our code is available on GitHub (https://github.com/papadomanolakiNM/NMP_UU_CO2P) through ZENODO (https://doi.org/10.5281/zenodo.5256597).
