# Supplementary material for: Eutrophication and Deoxygenation Forcing of Marginal Marine Organic Carbon Burial During the PETM
Source: Paleoceanogr Paleoclimatol. 2022 Mar 3;37(3):e2021PA004232. doi: 10.1029/2021PA004232 (PMC9310739; doi:10.1029/2021PA004232)
Supplement: Supplementary file 1 — Supporting Information S1 [file PALO-37-0-s003.pdf]

Paleoceanography and Paleoclimatology

Supporting Information for

Eutrophication and deoxygenation forcing of marginal marine organic carbon burial during the PETM

Nina M. Papadomanolaki, Appy Sluijs and Caroline P. Slomp

Department of Earth Sciences, Faculty of Geosciences, Utrecht University, Utrecht, the Netherlands

Contents of this file

Text S1 to S11
Figures S1 to S4
Tables ST2, ST5, ST6
PANGAEA DOI Information

Additional Supporting Information (Files uploaded separately)

Captions for Tables ST1, ST3, ST4, ST7, ST8
Captions for Dataset S1

Introduction

The Supplementary Text below provides additional information on the sites for which we generated new data and the method used to measure them. The new biogeochemical box model that is used in the paper is described in detail below, and additional simulations and sensitivity analyses are also presented. Additionally, calculations for the burial of organic carbon are presented. The figures support this information and the tables present the content of main text and supplementary figures, as well as additional model information. The new data is provided as dataset S1 and is also available on PANGAEA (see end of document).

S1. Site description

We generated new seafloor oxygenation proxy records for a selection of sites to fill gaps recognized in the compilation by Sluijs *et al*. (2014). These include the deep North Atlantic (International Ocean Discovery Program (IODP) Site 1403) and marginal settings in the Pacific,

Tethys, Atlantic, Arctic, Southern and Indian Oceans (Lodo Gulch, Forada, Bass River, Lomonosov Ridge IODP Site M004, Ocean Drilling Program (ODP) Sites 1172 and 752).

Site 1403 was drilled during IODP Expedition 342 and recovered the late Paleocene and Early Eocene off the Newfoundland (Norris *et al.*, 2014). The PETM was recognized by a negative $\delta^{13}C_{Corg}$ excursion and a break in carbonate sedimentation (Penman *et al.*, 2016). The interval corresponding to the PETM comprises $CaCO_3$-poor and $CaCO_3$-rich (nannofossil) claystones and was deposited at a paleodepth of ca. 4400 m (Penman *et al.*, 2016).

The Lodo Gulch section on the west coast of North America is comprised mostly of siltstone deposited at <200m water depth (Berggren and Aubert, 1983; John *et al.*, 2008). The CIE extends from approximately 6m to 20m and the onset and core of the CIE are marked by an increase in grain size, $CaCO_3$ and C_{org} (John *et al.*, 2008). The recovery interval is truncated by a glauconite-rich sand bed (John *et al.*, 2008).

The PETM at Forada spans ca. 5 m of clay, marl and limestone deposited at ca. 1000 m depth and is characterized by a $\delta^{13}C_{CaCO3}$ excursion between ~39.5m and 44m (Giusberti *et al.*, 2007). The core of the CIE occurs within a thick unit of clays and marls (the clay marl unit), which was the result of increased terrigenous flux to the site (Giusberti *et al.*, 2007).

Paleogene siliciclastic sediments with some biogenic carbonate at Bass River, New Jersey shelf (ODP Leg 174AX) were deposited close to the shelf edge (Miller *et al.*, 1998; Miller *et al.*, 2004). The PETM $\delta^{13}C_{CaCO3}$ excursion occurs in a clay-rich interval between ~347 mbsf and 357 mbsf (Cramer *et al.*, 1999; John *et al.*, 2008).

The upper Paleocene and lower Eocene green and gray clay- and siltstones at ODP Site 1172 are rich in organics but devoid of calcareous and siliceous microfossils and were deposited in a shallow marine environment (Shipboard Scientific Party, 2001; Röhl *et al.*, 2004). The thickness

of the CIE is between 65 and 90 cm, depending on the definition of the termination (Sluijs *et al.*, 2011).

Upper Paleocene to lower Eocene chalks, deposited at mid to lower bathyal depths, were recovered during ODP Expedition 121 Site 752 on the Broken Ridge in the Indian Ocean (Shipboard Scientific Party, 1989). The Paleocene-Eocene boundary is tentatively identified at 171.1 mbsf based on biostratigraphy and a negative shift in bulk carbonate $\delta^{13}C$ (Shipboard Scientific Party, 1989; Seto *et al.*, 1991).

S2. Geochemical analysis methods

Sediment samples were freeze-dried and powdered with an agate mortar and pestle. Between 100 and 125 mg of sample was weighed in Teflon destruction vessels, after which 2.5 ml concentrated mixed acid ($HClO_4$:HNO_3; 3:2) and 2.5 ml 40% HF were added. The mixture was then heated to 90°C and left overnight. Subsequently, the acids were evaporated at a temperature of 140°C, after which the residue was dissolved in 25 ml 4.5% HNO_3. Finally, the concentrations of iron (Fe), molybdenum (Mo), phosphorus (P) and aluminum (Al) were measured using Inductively Coupled Plasma-Optical Emission Spectrometry (ICP-OES) using a Perkin Elmer 9224 Optima 3000. Sedimentary Fe contents were normalized over Al. The relative standard deviation (RSD) calculated from duplicates for the elements presented in this study was generally ≤ 5%.

Approximately 300 mg of powdered freeze-dried sediment was weighed in centrifuge tubes for decalcification prior to organic carbon analysis. The samples were mixed twice with 7.5 ml 1 M HCl, shaken overnight and subsequently washed twice with 10 ml demineralized water (Van Santvoort *et al.*, 2002). Afterwards, the samples were dried in an oven at 50°C. Between 5 and 10 mg of decalcified sediment was weighed in tinfoil cups and analyzed using a Fisons Instruments CNS NA 1500 analyzer. Sedimentary Corg content was calculated after correcting the sediment weight for carbonate loss. Duplicate analyses set the RSD for Corg as ≤ 5%.

S3. New geochemical data

The $\delta^{13}C$ data for our newly generated PETM records show that we capture both the onset and recovery of the PETM at five out of seven sites (Supplementary Figure S1). Average C_{org} contents are mostly at or below ~0.5% at all sites except for ODP 1172 and the Lomonosov Ridge site where C_{org} contents range up to ~1.3 and 3.4 wt%, respectively. At this latter site, values for C_{org} decrease during the recovery phase, but remain high (>1%). At the remaining sites, fluctuations in C_{org} content show no discernible trend.

Values for C_{org}/P_{tot} are, likewise, low at most sites and generally below the Redfield ratio of 106. Average C_{org}/P_{tot} in excess of this value is only found at the Lomonosov Ridge while a maximum C_{org}/P_{tot} in excess of the Redfield value is only observed at this site and at Lodo Gulch. Although the C_{org}/P_{tot} is low, the increase with time observed at IODP Site 1403, Bass River and ODP Site 752 does suggest more reducing conditions during the PETM. Maxima in C_{org}/P_{tot} at Lodo Gulch and Forada may indicate brief deoxygenation. Increases in Fe/Al occur at IODP Site 1403, Lodo Gulch, Lomonosov Ridge and ODP Sites 1172 and 752. Molybdenum is present at Bass River, Lomonosov Ridge and ODP Sites 1172 and 752, with highest concentrations, up to 120 ppm, at the Lomonosov Ridge.

Five of our sediment records capture the recovery phase of the PETM. A minor increase in C_{org} and C_{org}/P_{tot} at IODP Site 1403 and a single peak in Fe/Al at Lodo Gulch occur during the recovery. At Bass River, only C_{org}/P_{tot} shows a change over the recovery, stabilizing at high values. Values of C_{org}, Fe/Al and Mo decrease during the recovery at the Lomonosov Ridge, but C_{org}/P_{tot} reaches its maximum value in the same interval, before eventually recovering as well. Similar to Bass River, Fe/Al at ODP Site 1172 stabilizes at its maximum value before declining, while C_{org}, C_{org}/P_{tot} and Mo show a minor increase across the entire recovery.

S4. Model water cycle

Dimensions for the open ocean boxes were derived from LOSCAR (Zeebe, 2012). Shallow boxes represent the mixed surface layer and have an average depth of either 150 or 200m (Supp. Table ST4). The thermocline box extends for 900m below the mid/low latitude surface ocean box. Assuming that the late Paleocene – early Eocene shelf area was approximately 1.5 – 2 times the size of the modern shelf (John *et al*., 2008), we set the total shelf size to 4.35 x 10^6 km^2. The Arctic Ocean was assigned an area of 0.3 x 10^6 km^2 (Sluijs *et al*., 2008a) and a total depth similar to the average depth of the modern basin (e.g. Abulaitijiang *et al*., 2019). The continental margin box was assigned an area twice (2.7 x 10^6 km^2) that of the EES (1.35 x 10^6 km^2) based on tectonic and sea level reconstructions for the Early Eocene (e.g. Seton *et al*., 2012; Miller *et al*., 2020). The deep section of the EES, with a depth of 750m, is represented by a separate box due to the uncertainty surrounding the depth of the EES and peri-Tethys, and the large number of sites in this area from a range of water depths (e.g., Gavrilov *et al*., 1997; 2003; Dickson *et al*., 2014).

The direction and value of the open ocean water fluxes were mostly based on LOSCAR (Zeebe, 2012) with remaining fluxes calculated by assuming steady state (Supp. Table ST5). Deep water formation in the open ocean is located in the Southern Ocean (e.g., Pak and Miller, 1992; Thomas *et al*., 2003), with formation cells in each of the three major deep basins (Atlantic, Indotethys, Pacific). Deep water exchange between the major deep ocean basins was most likely much restricted relative to the modern given Southern Ocean tectonic boundary conditions, but there likely was some (e.g., Zeebe and Zachos, 2007; Luo *et al*., 2016). We have not included any exchange in the model for simplicity. Upwelling is divided into coastal (80%) and equatorial (20%) upwelling. The upwelled water partially evaporates, returning as precipitation in the Southern Ocean, while the remainder flows from the margins, through the low latitudes to the Southern Ocean. The restricted Arctic and Epicontinental basins have limited surface exchange, also with the low latitude open ocean, and no deep water exchange with the open ocean (Fig. 2). Rivers flow into the Arctic, EES and open ocean marginal boxes at rates approximately proportional to the size of each basin; the Arctic receives a somewhat larger fraction (10%) than

dictated by its size (5%), as the modern Arctic makes up only 1% of the global seawater volume but receives 11% of global river outflow (Kalinin and Shiklomanov, 1974). The total riverine flux is increased relative to the modern value (Miliman and Farnsworth, 2013), assuming a stronger hydrological cycle for the late Paleocene (e.g. Pierrehumbert, 2002; Carmichael *et al.*, 2017). Precipitation and evaporation are added for steady state.

S5. Late Paleocene steady state for model

The simplified cycles of C, P and O_2 in our model include production, degradation and burial of organic matter (both C_{org} and P_{org}), as well as the burial of authigenic apatite (P_{auth}) and iron-bound P (P_{Fe}) (Supp. Table ST4). Terrestrial Corg and non-reactive, detrital P are not included. Total primary production in the model is ~48 Pg C yr^{-1}, which is close to the modern estimate for net primary production by coastal and oceanic phytoplankton (~54 Pg C yr^{-1}; Middelburg, 2019). We assume that production of P_{org} is linked to C_{org} through the Redfield ratio of 106:1, resulting in a total production of ~38 Tmol P yr^{-1}. Roughly 80% of the organic matter is produced in the low latitude ocean (S4) and Southern Ocean (S5), with the remainder occurring in the three marginal boxes (S1 – 3). In the modern ocean, the open ocean accounts for a somewhat higher proportion of total production (87%; Middelburg, 2019). In the warmer world of the late Paleocene (e.g. Cramwinckel *et al.*, 2018) with much higher sea level (e.g. Müller *et al.*, 2008; Miller *et al.*, 2020) production may have been shifted towards the shelves, as it did during the PETM itself (e.g. Gibbs *et al.*, 2006) and as projected for the future ocean (Bindoff *et al.*, 2019).

Approximately 92% of total C_{org} production is recycled in the surface ocean (modern ocean: ~80%; Middelburg, 2019). The percentage of organic carbon that decays in the surface boxes varies from 84% in S1 to 96% in S2. Export production is ~3.6 Pg C yr^{-1} (modern ocean: 1.6 – 3.4 Pg C yr^{-1}; Laws *et al.*, 2000a; 9 – 13 Pg C yr^{-1}; Laws *et al.*, 2011) and ~99% of the C_{org} is degraded in intermediate and deep boxes. Therefore, only 1% of export production (0.036 Pg C yr^{-1}) is buried in the sediments of the deep Arctic, EES, Atlantic, Indotethys and Pacific Oceans

(D1-5). A further 0.128 Pg C yr^{-1} is buried in the marginal boxes, in boxes S1 – 3, amounting to a total burial of ca. 0.3% of total primary production, or 0.16 Pg C yr^{-1} (Burdige, 2007).

In order to calculate the steady state burial fluxes for the three P phases, we set the initial $(C_{org}/P_{org})_{oxic}$ burial ratio for deep boxes to 260 (Komar and Zeebe, 2017), to 400 for the surface Arctic and EES (Slomp and Van Cappellen, 2007), and to 500 for the continental margin. The resulting C_{org}/P_{tot} ratios in our model are close to the Redfield value of 106 suggesting that we obtain a good approximation of reactive P burial in oxic marginal settings (Algeo and Ingall, 2007). Additionally, we assume that P_{org} burial is 25% of the total, P_{auth} is 50% and the remaining 25% is buried as P_{Fe} (Ruttenberg, 2003; Slomp and Van Cappellen, 2007). The total P_{org} and P_{Fe} burial in our model are 0.035 Tmol P yr^{-1} each, while P_{auth} is 0.07 Tmol P yr^{-1}. Therefore, total P burial in the global ocean amounts to 0.14 Tmol P yr^{-1}, which falls within the range assumed for reactive (i.e. biologically active) P burial in previous studies for the pre-anthropogenic ocean (e.g. 0.09 Tmol P yr^{-1}; Slomp and Van Cappellen, 2007; 0.30 Tmol yr^{-1}; Ruttenberg, 2003). The burial of P is balanced by riverine input. The resulting residence time for P in the ocean is ~11 kyr, which is at the lower end of the range typically considered (10 – 40 kyr) (Ruttenberg, 2003; Tsandev *et al.* 2008) and also lower than the residence time in LOSCAR (~40 kyr; Komar and Zeebe, 2017).

The degradation of organic matter consumes O_2 and we model this explicitly in the intermediate and deep boxes (IM, D1 – 5) as O_2 respiration. The steady state value for respiration is calculated using the corresponding value of Corg decay and the $(C/O_2)_{Redfield}$ value (Supp. Table ST6).

Dissolved P and O_2 are exchanged between all boxes. This transfer is directly linked to the water cycle and is equal to the product of the water flux and the concentration of the chemical constituent in the source box. Initial concentrations of dissolved P and O_2, the latter only for the intermediate and deep boxes, are calculated from the water flux and all other initial fluxes in the C, P and O_2 cycles, assuming steady state (Supp. Table ST4). Oxygen concentrations in surface waters depend on temperature and are calculated using the equations for the solubility of O_2

derived by Weiss (1970) for the modern ocean-atmosphere system, as in LOSCAR (Zeebe, 2012). The initial concentrations are comparable to those in the modern ocean (Garcia *et al.*, 2018a; 2018b).

S6. Model rate laws

In the dynamic version of the model, used to assess the effect of perturbations, the exchange of dissolved P and O_2 between boxes was calculated as the product of the water flux and the concentration of the dissolved constituent in the source box (see Slomp and Van Cappellen, 2007 and references therein). As an example, the upwelling of dissolved P from the deep Atlantic onto the continental margin is the product of the upwelling rate and the $[PO_4]$ of the deep Atlantic.

Assuming that P is the limiting nutrient on geological timescales (e.g. Tyrrell, 1999), the formation of P_{org} through primary production in each surface box is dependent on $[PO_4]$ and a rate constant (Supp. Table ST6):

$$PP = [PO_4] \times k_{bio} \tag{1}$$

A set fraction f_{exp} of primary production is exported directly to the deep ocean (from S1, S3, S5) or to the thermocline (from S2, S4). Primary production and export of C_{org} are linked to P_{org} through the Redfield ratio. In all boxes, the fraction of C_{org} and P_{org} that is not exported (S1 – 5; IM) or buried (S1 – 3; D1 – 5) is remineralized.

In the thermocline and deep ocean, the remineralization of C_{org} consumes O2 at the Redfield ratio of 106:138 (Supp. Table ST6). The additional effects of stratification and C_{org} degradation on deoxygenation at the sediment – water interface are captured in the degree of anoxia (DOA) (Van Cappellen and Ingall, 1994). A value of zero describes fully oxic conditions while a value of 1 is linked to complete anoxia:

$$DOA = 1 - \left(\frac{f_{strat} \times [O_2]}{[O_2]_0} \right) \times \left(\frac{PP_0}{PP} \right)$$

(2)

where PP_0 is the steady state value for primary production and PP is primary production as calculated in (1). The term f_{strat} captures the effect of increased water column stratification as a result of rising temperatures and reduced salinity. A value of 1 implies a lack of stratification, whereas a value of zero is assigned when there is no vertical exchange within the water column of a box, hence stratification is complete. In complex models, this effect is described by a vertical diffusion coefficient which is generally dependent on density and ocean floor topography (e.g. Bouttes *et al*., 2009). Such complexities are, however, beyond the capabilities of box models such as ours. We therefore assign a time dependent increase to a maximum value within the plateau of the PETM, and a time dependent linear decrease back to 1 during the recovery.

In the marginal boxes (S1 – 3), a fraction of P is buried in sediments in the form of P_{org}, P_{auth} and P_{Fe}. The burial rates for all three phases are dependent on the degree of anoxia (DOA) in each box. Burial of P_{org} depends on the rate of primary production (PP), a rate constant (k_{orgP}) and DOA, assuming that only a fraction f_{orgP} of P_{org} burial is redox-dependent:

$$P_{org} = k_{orgP} \times PP \times \left(\left(1 - f_{orgP}\right) + f_{orgP} \times (1 - DOA) \right)$$

(3)

The burial rate of P_{auth} depends on the remineralization of organic matter (R), a rate constant (k_{authP}) and DOA assuming a redox dependent fraction f_{authP}:

$$P_{auth} = k_{authP} \times R \times \left(\left(1 - f_{authP}\right) + f_{authP} \times (1 - DOA) \right)$$

(4)

Burial of P_{Fe} depends linearly on DOA:

$$P_{Fe} = P_{Fe,0} \times (1 - DOA) \tag{5}$$

The burial rate of C_{org} is linked to that of P_{org} through the C_{org}/P_{org} burial ratio, with a value that depends on bottom water redox conditions (Van Cappellen and Ingall, 1994):

$$C_{org} = P_{org} \times \frac{C/P_{oxic} \times C/P_{anoxic}}{C/P_{anoxic} \times (1 - DOA) + C/P_{oxic} \times (DOA)}$$

(6)

In the deep ocean, burial of P_{org}, P_{auth}, P_{Fe} and C_{org} are modelled in a similar manner but here they depend on O_2 concentrations instead of DOA. Furthermore, P_{org} burial depends on P export rather than productivity:

$$P_{org} = k_{orgP} \times E \times \left(\left(1 - f_{orgP}\right) + f_{orgP} \times \left(\frac{[O_2]}{[O_2]_0} \right) \right)$$

(7)

$$P_{auth} = k_{authP} \times R \times \left(\left(1 - f_{authP}\right) + f_{authP} \times \left(\frac{[O_2]}{[O_2]_0} \right) \right)$$

(8)

$$P_{Fe} = P_{Fe,0} \times \left(\frac{[O_2]}{[O_2]_0}\right)$$

(9)

$$C_{org} = P_{org} \times \frac{C/P_{oxic} \times C/P_{anoxic}}{C/P_{anoxic} \times \left(\frac{[O_2]}{[O_2]_0}\right) + C/P_{oxic} \times \left(1 - \frac{[O_2]}{[O_2]_0}\right)}$$

(10)

Weathering of silicate and carbonate rocks on land is linked to atmospheric pCO_2 and provides reactive P to the ocean through rivers. The response of weathering of carbonates (W_{carb}) and silicates (W_{sil}) to changes in pCO_2 is modelled as in LOSCAR, by assuming a non-linear dependence on a factor n_{cc} and n_s, respectively:

$$W_{carb} = W_{carb,0} \times \frac{pCO_2}{pCO_{2,0}}^{n_{cc}}$$

(13)

$$W_{sil} = W_{sil,0} \times \frac{pCO_2}{pCO_{2,0}}^{n_s}$$

(14)

The response of P weathering (P_{weath}), in turn, depends on carbonate and silicate weathering modulated by a factor n_p :

$$P_{weath} = (P_{weath,0} \times \frac{W_{carb} + W_{sil}}{W_{carb,0} + W_{sil,0}})^{n_p}$$

(15)

The riverine flux of P (P_{in}) to each marginal box (S1-S3) is a function of P_{weath} and of its coastline and area and an estimation of the number of rivers draining into the region, which are combined in the factor fin:.

$$P_{in} = P_{weath} \times f_{in} \tag{16}$$

Parameter values are either taken from previous publication (e.g. Komar and Zeebe, 2017) or calculated from steady state values of the fluxes and concentrations and their corresponding rate laws. Key parameter values can be found in Supp. Table ST6.

A key output of the model is the excess burial of C_{org}: the amount of C_{org} buried in addition to the burial mass of late Paleocene (steady state) conditions. Excess C_{org} burial for a given time-step is the difference between the C_{org} burial rate at that time-step and the steady state burial rate, times the duration of the time-step. By adding the excess buried mass for all time-steps within a certain interval of the PETM (e.g. the recovery), we obtain the excess C_{org} burial for that interval. Other excess mass values, such as riverine P influx (Fig. 3) and primary production are calculated in a similar manner.

S7. Additional model simulations

The standard simulation presented in the main text is forced by the increased weathering of phosphorus (P), as a result of increased CO_2 following the curve presented by Zeebe *et al*. (2009) (Z09). We tested the biogeochemical response to three more CO_2 curves and present them here. Two of the curves are published by Frieling *et al*. (2016) (F16) and Gutjahr *et al*. (2017) (G17) whereas the third is an adjusted version of the Zeebe *et al*. (2009) curve with a 170 kyr duration for the stable isotope phase (Zeebe and Lourens, 2019) (K170). The forcing is, as in the standard Z09 simulation, an increase in weathering of P (Supp. Fig. S3A) which is then supplied to the marginal boxes (S1 – 3) of our model.

The results for the three additional simulations are very similar to those of the standard Z09 simulation, as evidenced by excess C_{org} burial during the full event and the first 40kyr of the recovery (Supp. Fig. S3B; see also Supp. Tables ST3, ST7). The similarities are largely due to the

similar magnitude of the CO_2 change for the four different simulations. The different shapes of the CO_2 curves, in particular those of Frieling *et al*. (2016) and Gutjahr *et al*. (2017), result in a slight increase in excess C_{org} burial. The main difference is in the higher overall excess burial for K170, versus that of Z09, F16 and G17 (Supp. Fig. S3B). This is due to the longer duration of the stable $\delta^{13}C$ phase of this scenario, giving more time for enhanced C_{org} burial to occur over.

S8. Redox sensitivity of Porg and Pauth burial

A key biogeochemical feedback driving C_{org} burial is the increase in redox-driven P recycling (see main text). In turn, the model response of P recycling to deoxygenation depends on the assigned sensitivity. The change in iron-bound P (P_{Fe}) burial due to deoxygenation is usually modelled as a linear relationship (Ingall and Van Cappellen, 1994). In the standard equations for organic P (P_{org}) and authigenic P (P_{auth}) burial (1, 2), the sensitivity is denoted as f_{OrgP} and f_{CaP}:

$$P_{org} = k_{orgP} \times PP \times \left(\left(1 - f_{orgP} \right) + f_{orgP} \times (1 - DOA) \right)$$

(1)

$$P_{auth} = k_{authP} \times R \times \left(\left(1 - f_{authP} \right) + f_{authP} \times (1 - DOA) \right)$$

(2)

Different values have been given to these two factors across a number of publications. In a study of the sensitivity of P cycling to ocean circulation in a model for the modern ocean (Slomp and Van Cappellen, 2007), the values for f_{OrgP} and f_{auth} were set at 0.25 and 0.5, respectively. Ruvalcaba Baroni *et al*. (2014), while modelling biogeochemical changes during Oceanic Anoxic Event 2 (OAE2), used an f_{OrgP} value of 0.4, a coastal f_{auth} value of 0.82 and an open ocean f_{auth} value of 0.18. Again for OAE2, Tsandev and Slomp (2009) use values of 0.75 and 0.9 for f_{OrgP} and f_{auth}, respectively. These last two values were also used by Komar and Zeebe (2017) for PETM simulations.

The choice of P burial sensitivity depends on the design of the model and the sensitivity of the C_{org}/P_{tot} ratio to anoxia inferred for the time period from the geological record or other considerations. For example, the higher values used by Ruvalcaba Baroni *et al*. (2014) and Tsandev and Slomp (2009) are explained by the severe and widespread nature of OAE2 deoxygenation (e.g. Takashima, 2004) and the reported high values of C_{org}/P_{tot} ratios (up to 2000). To recreate these conditions in a model requires intense P recycling relative to C_{org} (Ruvalcaba Baroni *et al*., 2014). On the other hand, the study of Komar and Zeebe (2017) focusses on C_{org} burial in the deep ocean which, with late Paleocene circulation, does not become hypoxic easily. As a result, intense P recycling is required to obtain the target value of excess C_{org} burial and the corresponding $\delta^{13}C$ curve.

In this study, we chose values of 0.75 for f_{Org} (Tsandev and Slomp, 2009) and 0.4 for f_{auth}, which is closer to the Slomp and Van Cappellen (2007) value than the high Komar and Zeebe (2017) value. The lower sensitivity for P_{auth} when compared to early studies is in line with milder deoxygenation during the PETM, compared to OAE2 (e.g. Jenkyns, 2010). These values resulted in excess C_{org} burial and relative C_{org}/P_{tot} changes in line with the data and previous publications. We also tested the response of our model to different values of f_{OrgP} and f_{auth}, using the Z09 forcing scenario, under strong (including increased stratification) and weak (without increased stratification) deoxygenation (Supp. Fig. S4A). A higher value of f_{auth} and stronger deoxygenation both cause an increase in excess C_{org} burial, as they promote P recycling and primary productivity. The response to increasing f_{OrgP} is opposite which appears counterintuitive. As P_{org} recycling gets stronger (higher f_{OrgP}), the supply of new organic matter is not sufficient to counteract the decreasing P_{org} burial. This effect is further strengthened under strong deoxygenation. The degree of deoxygenation also controls the value of C_{org}/P_{org} and as P_{org} gets lower at higher sensitivity, the corresponding C_{org} is also reduced. In all cases, C_{org} burial will still be higher than the late Paleocene value.

S9. Model stratification sensitivity

Sluijs *et al*. (2006), among others, showed that stratification increased during the PETM, likely as a result of an enhanced hydrological cycle (e.g. Carmichael *et al*., 2017). Stratification can be a key cause for deoxygenation in marginal marine areas and for this reason we have parameterized it in our model by adding a factor f_{strat} to the DOA for the surface Arctic and EES boxes. The Arctic Ocean and EES experienced severe deoxygenation, even anoxia (e.g. Sluijs *et al*., 2008; Dickson *et al*., 2014), which our model did not simulate without the addition of f_{strat}. We chose to use an exponential increase in f_{strat}, to match the shape of the increase in DOA without f_{strat} (Supp. Fig. 4B). As stratification is linked to a warmer, wetter climate due to rising pCO_2, we force a recovery of f_{strat} back to 1 (no increased stratification) at the beginning of the PETM recovery phase (Supp. Fig. 4B).

The value of DOA is approximately the same for all three marginal boxes, when increased stratification is not enforced (Supp. Table ST3). The relationship between f_{strat} and DOA is broadly linear (Supp. Fig. 4C) and the effect of f_{strat} is the same, regardless of the box it is enforced on. This is evident when comparing the maximum DOA value of the surface Arctic (Supp. Fig. 4C) and the surface EES (Fig. 6) for an f_{strat} of 0.4. We chose to use a lower f_{strat} value for the surface EES (0.4), compared to the surface Arctic (0.1), resulting in a lower DOA for the EES.

S10. Organic carbon burial across the PETM

To calculate C_{org} burial from the available TOC records for the PETM, we first have to calculate sedimentation rates for all available records. Studies on multiple locations for the PETM show an increase in MAR from the stable isotope phase to the PETM recovery (e.g. John *et al*., 2008; Dunkley Jones *et al*., 2018). For most sites, there are no (detailed) age models available to calculate changes in MAR within the event. For this reason, we calculate an average sedimentation rate for each site, using the thickness of the TOC record and two average durations

of 170 kyr (short PETM) and 290 kyr (long PETM; Zeebe and Lourens, 2019). Similar to Owens *et al*. (2018), we use a constant rock density of 2.4 g/cm^3 for sediments with a high average TOC content (>2%wt) and a rock density of 2.7 g/cm^3 for the other sediments. Our sites are grouped into four environments (shelf/slope, Arctic ocean, European Epicontinental Seaway and Deep open ocean) and we use the area used in our model for each of these to calculate total burial over the PETM.

Using the average MAR values for each environment results in a total burial of ~14,000 Pg C for the short PETM and 10,800 Pg C for the long PETM (Supp. Table ST8). Maximum MAR values result in a burial of 26,300 Pg C across the short PETM and of 23,000 Pg C across the long PETM. For the entire recovery interval (120 kyr), average MAR values result in a burial of ~10,000 Pg C during the short PETM and of 4500 Pg C during the long PETM. The same burial values for maximum MAR are 18,500 Pg C for the short PETM and 9500 Pg C for the long PETM. These values are lower than the total burial simulated by our model for the recovery phase for the short PETM (~33,000 Pg C). As noted in the main text, it is possible that our model overestimates the amount of burial occurring in the later part of the recovery. Average MAR may also underestimate the burial that occurred during the recovery phase by not accounting for temporal changes within the PETM.

S11. References Supplementary Materials

Abulaitijiang, A., Andersen, O. B., & Sandwell, D. (2019). Improved Arctic Ocean bathymetry derived from DTU17 gravity model. *Earth and Space Science*, 6(8), 1336-1347.

Algeo, T. J., & Ingall, E. (2007). Sedimentary Corg: P ratios, paleocean ventilation, and Phanerozoic atmospheric pO_2. *Palaeogeography, Palaeoclimatology, Palaeoecology*, 256(3-4), 130-155.

Berggren, W. A., & Aubert, J. (1983). Paleogene benthonic foraminiferal biostratigraphy and bathymetry of the Central Coast Ranges of California.

Bindoff, N.L., W.W.L. Cheung, J.G. Kairo, J. Arístegui, V.A. Guinder, R. Hallberg, N. Hilmi, N. Jiao, M.S. Karim, L. Levin, S. O'Donoghue, S.R. Purca Cuicapusa, B. Rinkevich, T. Suga, A. Tagliabue, and P. Williamson, 2019: Changing Ocean, Marine Ecosystems, and Dependent Communities. *In*: IPCC 2019: *Special Report on the Ocean and Cryosphere in a Changing Climate* [H.-O. Pörtner, D.C. Roberts, V. Masson-Delmotte, P. Zhai, M. Tignor, E. Poloczanska, K. Mintenbeck, A. Alegría, M. Nicolai, A. Okem, J. Petzold, B. Rama, N.M. Weyer (eds.)].

Bouttes, N., Roche, D. M., & Paillard, D. (2009). Impact of strong deep ocean stratification on the glacial carbon cycle. *Paleoceanography*, 24(3).

Burdige, D. J. (2007). Preservation of organic matter in marine sediments: controls, mechanisms, and an imbalance in sediment organic carbon budgets?. *Chemical reviews*, 107(2), 467-485.

Carmichael, M. J., Inglis, G. N., Badger, M. P., Naafs, B. D. A., Behrooz, L., Remmelzwaal, S., ... & Dickson, A. J. (2017). Hydrological and associated biogeochemical consequences of rapid global warming during the Paleocene-Eocene Thermal Maximum. *Global and Planetary Change*, 157, 114-138.

Cramer, B. S., Aubry, M. P., Miller, K. G., Olsson, R. K., Wright, J. D., & Kent, D. V. (1999). An exceptional chronologic, isotopic, and clay mineralogic record of the latest Paleocene thermal maximum, Bass River, NJ, ODP 174AX. *Bulletin de la Société géologique de France*, 170(6), 883-897.

Cramwinckel, M. J., Huber, M., Kocken, I. J., Agnini, C., Bijl, P. K., Bohaty, S. M., ... & Peterse, F. (2018). Synchronous tropical and polar temperature evolution in the Eocene. *Nature*, 559(7714), 382-386.

Dickson, A. J., Rees‐Owen, R. L., März, C., Coe, A. L., Cohen, A. S., Pancost, R. D., ... & Shcherbinina, E. (2014). The spread of marine anoxia on the northern Tethys margin during the Paleocene‐Eocene Thermal Maximum. *Paleoceanography*, 29(6), 471-488.

Frieling, J., Svensen, H. H., Planke, S., Cramwinckel, M. J., Selnes, H., & Sluijs, A. (2016). Thermogenic methane release as a cause for the long duration of the PETM. *Proceedings of the National Academy of Sciences*, 113(43), 12059-12064.

Garcia, H. E., K. Weathers, C. R. Paver, I. Smolyar, T. P. Boyer, R. A. Locarnini, M. M. Zweng, A. V. Mishonov, O. K. Baranova, D. Seidov, and J. R. Reagan (2018). World Ocean Atlas 2018, Volume 3: Dissolved Oxygen, Apparent Oxygen Utilization, and Oxygen Saturation. *A. Mishonov Technical Ed.; NOAA Atlas NESDIS*, 83, 38 pp.

Garcia, H. E., K. Weathers, C. R. Paver, I. Smolyar, T. P. Boyer, R. A. Locarnini, M. M. Zweng, A. V. Mishonov, O. K. Baranova, D. Seidov, and J. R. Reagan (2018). World Ocean Atlas 2018, Volume 4: Dissolved Inorganic Nutrients (phosphate, nitrate and nitrate+nitrite, silicate). *A. Mishonov Technical Ed.; NOAA Atlas NESDIS*, 84, 35 pp.

Gavrilov, Y. O., Kodina, L. A., Lubchenko, I. Y., & Muzylev, N. G. (1997). The late Paleocene anoxic event in epicontinental seas of Peri-Tethys and formation of the sapropelite unit: Sedimentology and geochemistry. *Lithology and Mineral Resources C/C of Litologiia I Poleznye Iskopaemye* 32, 427-450.

Gavrilov, Y. O., Shcherbinina, E. A., & Oberhansli, H. (2003). Paleocene-Eocene boundary events in the northeastern Peri-Tethys. *Special Papers – Geological Society of America*, 147-168.

Gibbs, S. J., Bralower, T. J., Bown, P. R., Zachos, J. C., & Bybell, L. M. (2006). Shelf and open-ocean calcareous phytoplankton assemblages across the Paleocene-Eocene Thermal Maximum: Implications for global productivity gradients. *Geology*, 34(4), 233-236.

Giusberti, L., Rio, D., Agnini, C., Backman, J., Fornaciari, E., Tateo, F., & Oddone, M. (2007). Mode and tempo of the Paleocene-Eocene thermal maximum in an expanded section from the Venetian pre-Alps. *GSA Bulletin*, 119(3-4), 391-412.

Gutjahr, M., Ridgwell, A., Sexton, P. F., Anagnostou, E., Pearson, P. N., Pälike, H., ... & Foster, G. L. (2017). Very large release of mostly volcanic carbon during the Palaeocene–Eocene Thermal Maximum. *Nature*, 548(7669), 573-577.

Jenkyns, H. C. (2010). Geochemistry of oceanic anoxic events. *Geochemistry, Geophysics, Geosystems*, 11(3).

John, C. M., Bohaty, S. M., Zachos, J. C., Sluijs, A., Gibbs, S., Brinkhuis, H., & Bralower, T. J. (2008). North American continental margin records of the Paleocene−Eocene thermal maximum: Implications for global carbon and hydrological cycling. *Paleoceanography*, 23(2).

Kalinin, G.P., and I.A. Shiklomanov (1974). Exploitation of the Earth's water resources, in World Water Balance and Water Resources of the Earth (in Russian), pp. 575-606, Gidrometeoizdat, St. Petersburg. Russia.

Komar, N., & Zeebe, R. E. (2017). Redox-controlled carbon and phosphorus burial: A mechanism for enhanced organic carbon sequestration during the PETM. *Earth and Planetary Science Letters*, 479, 71-82.

Laws, E. A., Landry, M. R., Barber, R. T., Campbell, L., Dickson, M. L., & Marra, J. (2000a). Carbon cycling in primary production bottle incubations: inferences from grazing experiments and photosynthetic studies using 14C and 18O in the Arabian Sea. *Deep Sea Research Part II: Topical Studies in Oceanography*, 47(7-8), 1339-1352.

Laws, E. A., D'Sa, E., & Naik, P. (2011). Simple equations to estimate ratios of new or export production to total production from satellite−derived estimates of sea surface temperature and primary production. *Limnology and Oceanography: Methods*, 9(12), 593-601.

Luo, Y., Boudreau, B. P., Dickens, G. R., Sluijs, A., & Middelburg, J. J. (2016). An alternative model for CaCO3 over-shooting during the PETM: biological carbonate compensation. *Earth and Planetary Science Letters*, 453, 223-233.

Middelburg, J. J. (2019). Marine carbon biogeochemistry: a primer for earth system scientists (p. 118). Springer Nature.

Milliman, J. D., & Farnsworth, K. L. (2013). River discharge to the coastal ocean: a global synthesis. Cambridge University Press.

Miller, K. G., Sugarman, P. J., Browning, J. V., Olsson, R. K., Pekar, S. F., Reilly, T. J., ... & Stewart, M. (1998). Bass river site. In Miller, KG, Sugarman, PJ, Browning, JV, et al., *Proc. ODP, Init. Repts., 174AX*: College Station, TX (Ocean Drilling Program) (pp. 5-43).

Miller, K. G., Sugarman, P. J., Browning, J. V., Kominz, M. A., Olsson, R. K., Feigenson, M. D., & Hernández, J. C. (2004). Upper Cretaceous sequences and sea-level history, New Jersey coastal plain. *GSA Bulletin*, 116(3-4), 368-393.

Miller, K. G., Browning, J. V., Schmelz, W. J., Kopp, R. E., Mountain, G. S., & Wright, J. D. (2020). Cenozoic sea-level and cryospheric evolution from deep-sea geochemical and continental margin records. *Science advances*, 6(20), 1346.

Müller, R. D., Sdrolias, M., Gaina, C., Steinberger, B., & Heine, C. (2008). Long-term sea-level fluctuations driven by ocean basin dynamics. *Science*, 319(5868), 1357-1362.

Norris, R.D., Wilson, P.A., Blum, P., Fehr, A., Agnini, C., Bornemann, A., Boulila, S., Bown, P.R., Cournede, C., Friedrich, O., Ghosh, A.K., Hollis, C.J., Hull, P.M., Jo, K., Junium, C.K., Kaneko, M., Liebrand, D., Lippert, P.C., Liu, Z., Matsui, H., Moriya, K., Nishi, H., Opdyke, B.N., Penman, D., Romans, B., Scher, H.D., Sexton, P., Takagi, H., Turner, S.K., Whiteside, J.H., Yamaguchi, T., and Yamamoto, Y., 2014. Site U1403. *In* Norris, R.D., Wilson, P.A., Blum, P., and the Expedition 342 Scientists, *Proc. IODP, 342*: College Station, TX (Integrated Ocean Drilling Program).

Pak, D. K., & Miller, K. G. (1992). Paleocene to Eocene benthic foraminiferal isotopes and assemblages: Implications for deepwater circulation. *Paleoceanography*, 7(4), 405-422.

Penman, D. E., Turner, S. K., Sexton, P. F., Norris, R. D., Dickson, A. J., Boulila, S., ... & Westerhold, T. (2016). An abyssal carbonate compensation depth overshoot in the aftermath of the Palaeocene–Eocene Thermal Maximum. *Nature Geoscience*, 9(8), 575-580.

Pierrehumbert, R. T. (2002). The hydrologic cycle in deep-time climate problems. *Nature*, 419(6903), 191-198.

Röhl, U., Brinkhuis, H., Fuller, M. D., Schellenberg, S. A., Stickley, C. E., & Williams, G. L. (2004). Cyclostratigraphy of middle and late Eocene sediments drilled on the East Tasman Plateau (Site 1172). *In* Exon, N.F., Kennett, J.P., and Malone, M.J. (Eds.), The Cenozoic Southern Ocean: Tectonics, Sedimentation and Climate Change between Australia and Antarctica. *Am. Geophys. Union, Geophys. Monogr*., 151:127-151.

Ruttenberg, K. C. (2003). The global phosphorus cycle. *Treatise on Geochemistry*, 8, 682.

Ruvalcaba Baroni, I., Topper, R. P. M., Van Helmond, N. A. G. M., Brinkhuis, H., & Slomp, C. P. (2014). Biogeochemistry of the North Atlantic during oceanic anoxic event 2: role of changes in ocean circulation and phosphorus input. *Biogeosciences*, 11(4), 977-993.

Seto, K., Nomura, R., and Niitsuma, N., 1991. Data report: oxygen and carbon isotope records of the upper Maestrichtian to lower Eocene benthic foraminifers at Site 752 in the eastern Indian Ocean. *In* Weissel, J., Peirce, J., Taylor, E., Alt, J., et al., *Proc. ODP, Sci. Results*, 121: College Station, TX (Ocean Drilling Program), 885–889.

Seton, M., Müller, R. D., Zahirovic, S., Gaina, C., Torsvik, T., Shephard, G., ... & Chandler, M. (2012). Global continental and ocean basin reconstructions since 200 Ma. *Earth-Science Reviews*, 113(3-4), 212-270.

Shipboard Scientific Party, 1989. Site 752. *In* Peirce, J., Weissel, J., et al., *Proc. ODP, Init. Repts*., 121: College Station, TX (Ocean Drilling Program), 111–169.

Shipboard Scientific Party, 2001. Site 1172. *In* Exon, N.F., Kennett, J.P., Malone, M.J., et al., *Proc. ODP, Init. Repts., 189*: College Station TX (Ocean Drilling Program), 1–98.

Slomp, C. P., & Cappellen, P. V. (2007). The global marine phosphorus cycle: sensitivity to oceanic circulation. *Biogeosciences*, 4(2), 155-171.

Sluijs, A., Schouten, S., Pagani, M., Woltering, M., Brinkhuis, H., Damsté, J. S. S., ... & Matthiessen, J. (2006). Subtropical Arctic Ocean temperatures during the Palaeocene/ Eocene thermal maximum. *Nature*, 441(7093), 610-613.

Sluijs, A., Röhl, U., Schouten, S., Brumsack, H. J., Sangiorgi, F., Damsté, J. S. S., & Brinkhuis, H. (2008a). Arctic late Paleocene–early Eocene paleoenvironments with special emphasis

on the Paleocene−Eocene thermal maximum (Lomonosov Ridge, Integrated Ocean Drilling Program Expedition 302). *Paleoceanography*, 23(1).

Sluijs, A., van Roij, L., Harrington, G. J., Schouten, S., Sessa, J. A., LeVay, L. J., ... & Slomp, C. P. (2014). Warming, euxinia and sea level rise during the Paleocene-Eocene Thermal Maximum on the Gulf Coastal Plain: implications for ocean oxygenation and nutrient cycling. *Climate of the Past*, 10(4), 1421-1421.

Stassen, P., Thomas, E., & Speijer, R. P. (2012). Integrated stratigraphy of the Paleocene−Eocene thermal maximum in the New Jersey Coastal Plain: Toward understanding the effects of global warming in a shelf environment. *Paleoceanography*, 27(4).

Thomas, D. J., Bralower, T. J., & Jones, C. E. (2003). Neodymium isotopic reconstruction of late Paleocene–early Eocene thermohaline circulation. *Earth and Planetary Science Letters*, 209(3-4), 309-322.

Tsandev, I., Slomp, C. P., & Van Cappellen, P. (2008). Glacial−interglacial variations in marine phosphorus cycling: Implications for ocean productivity. *Global Biogeochemical Cycles*, 22(4).

Tsandev, I., & Slomp, C. P. (2009). Modeling phosphorus cycling and carbon burial during Cretaceous Oceanic Anoxic Events. *Earth and Planetary Science Letters*, 286(1-2), 71-79.

Tyrrell, T. (1999). The relative influences of nitrogen and phosphorus on oceanic primary production. *Nature*, 400(6744), 525-531.

Van Cappellen, P., & Ingall, E. D. (1994). Benthic phosphorus regeneration, net primary production, and ocean anoxia: a model of the coupled marine biogeochemical cycles of carbon and phosphorus. Paleoceanography, 9(5), 677-692.

Van Santvoort, P. J. M., De Lange, G. J., Thomson, J., Colley, S., Meysman, F. J. R., & Slomp, C. P. (2002). Oxidation and origin of organic matter in surficial Eastern Mediterranean hemipelagic sediments. *Aquatic Geochemistry*, 8(3), 153-175.

Weiss, R. F. (1970). The solubility of nitrogen, oxygen and argon in water and seawater. *In* Deep sea research and oceanographic abstracts, 17(4),721-735.

Zeebe, R. E., & Zachos, J. C. (2007). Reversed deep−sea carbonate ion basin gradient during Paleocene−Eocene thermal maximum. *Paleoceanography*, 22(3).

Zeebe, R. E., Zachos, J. C., & Dickens, G. R. (2009). Carbon dioxide forcing alone insufficient to explain Palaeocene–Eocene Thermal Maximum warming. *Nature Geoscience*, 2(8), 576-580.

Zeebe, R. E. (2012). LOSCAR: Long-term ocean-atmosphere-sediment carbon cycle reservoir model v2. 0.4. *Geoscientific Model Development*, 5(1), 149.

Zeebe, R. E., & Lourens, L. J. (2019). Solar System chaos and the Paleocene–Eocene boundary age constrained by geology and astronomy. *Science*, 365(6456), 926-929.

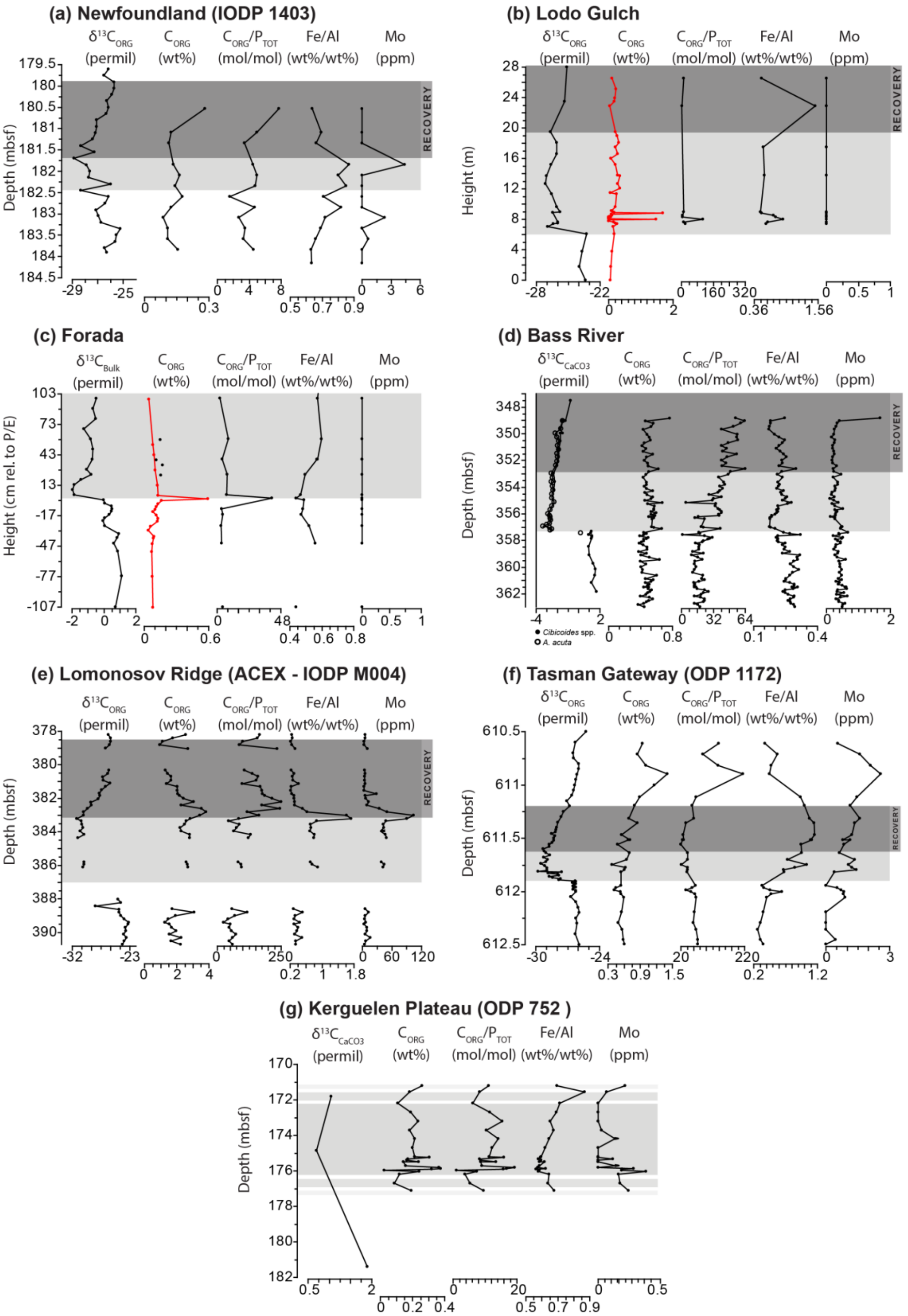

Figure S1. PETM δ13C stratigraphy with bulk geochemical data: total organic carbon content (C_{org}), C_{org}/P_{tot}, Fe/Al Mo concentrations. δ13C records are from Penman *et al.*, 2016 (IODP 1403), John *et al.*, 2008 (Lodo Gulch), Giusberti *et al.*, 2007 (Forada), Cramer *et al.*, 1999 (Bass River), Sluijs *et al.*, 2006 (Lomonosov Ridge), Sluijs *et al.*, 2011 (ODP 1172) and Seto *et al.*, 1991 (ODP 752). Organic carbon content for Lodo Gulch and Forada (red) are from John *et al.*, 2008 and Giusberti *et al.*, 2007, respectively. Grey bars indicate the extent of the PETM. Dark

bars indicate the presumed extent for the recovery phase in each record (recovery phase I at Bass River, Stassen *et al*., 2012). The lighter bands for ODP 752 indicate the uncertainty in the extent of the PETM.

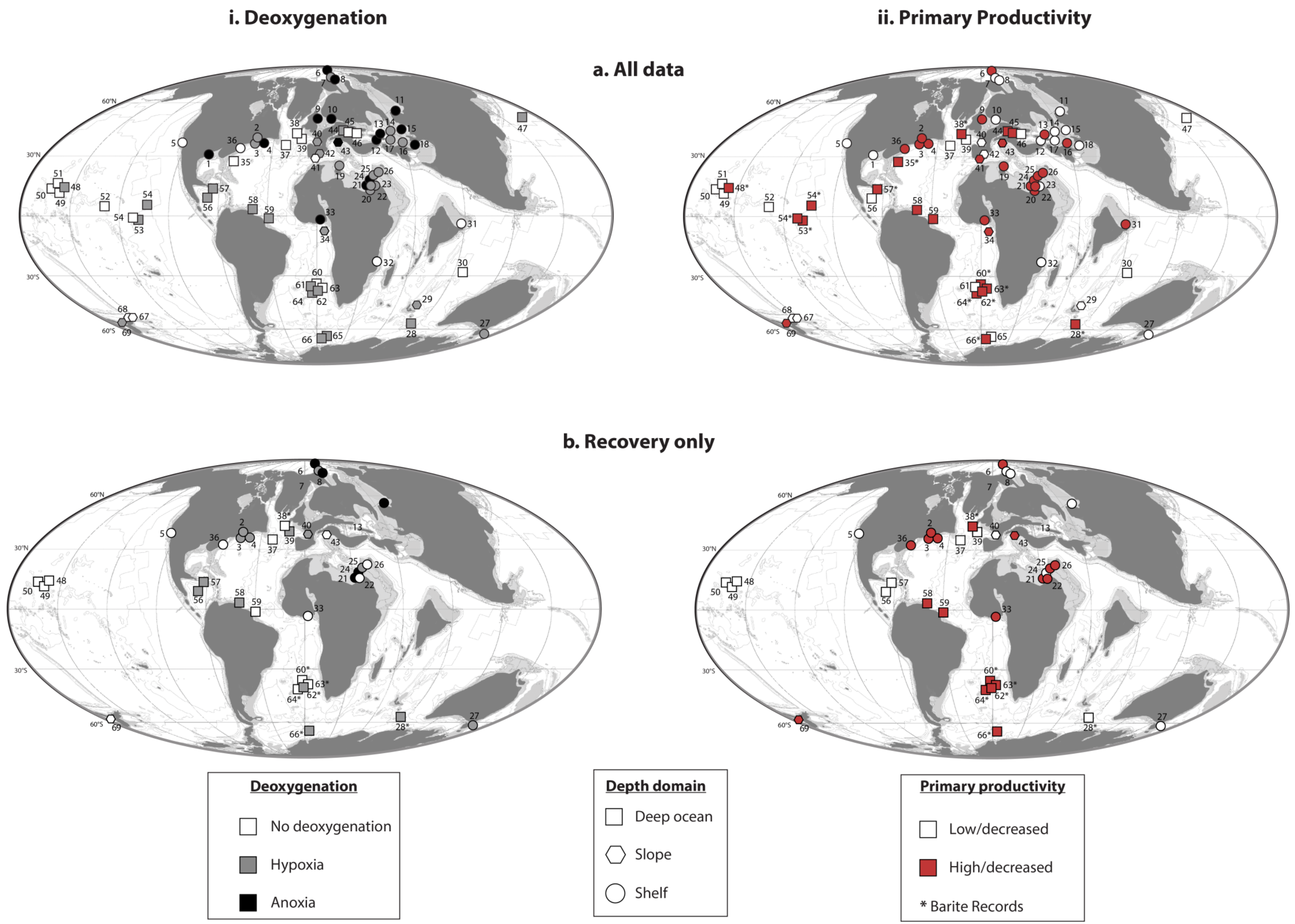

i. Deoxygenation
ii. Primary Productivity
a. All data
b. Recovery only
Deoxygenation
No deoxygenation
Hypoxia
Anoxia
Depth domain
Deep ocean
Slope
Shelf
Primary productivity
Low/decreased
High/decreased
* Barite Records

Figure S2. Global overview of sites of proxy records on changes in bottom water redox conditions (i) primary productivity (PP) (ii) for the PETM. Records for all phases of the PETM (a) and records for the recovery as identified from the $\delta^{13}C$ excursion (b) are indicated separately. For the redox conditions, white indicates oxic conditions or no available data, grey indicates evidence for hypoxia and black indicates evidence for anoxia and/or euxinia. For primary productivity, white is used for sites with no data on productivity or low/decreased productivity. Red is used for sites with evidence for high/increased productivity. Sites with records that use barite to reconstruct PP are indicated by stars (*). Symbol shapes represent the depth domain: shelf (circle), slope (polygon) and deep (square). For the full reference list and site names (here indicated by numbers) see Supp. Table ST1. Map after Markwick (2007), modified by Sluijs *et al*. (2014).

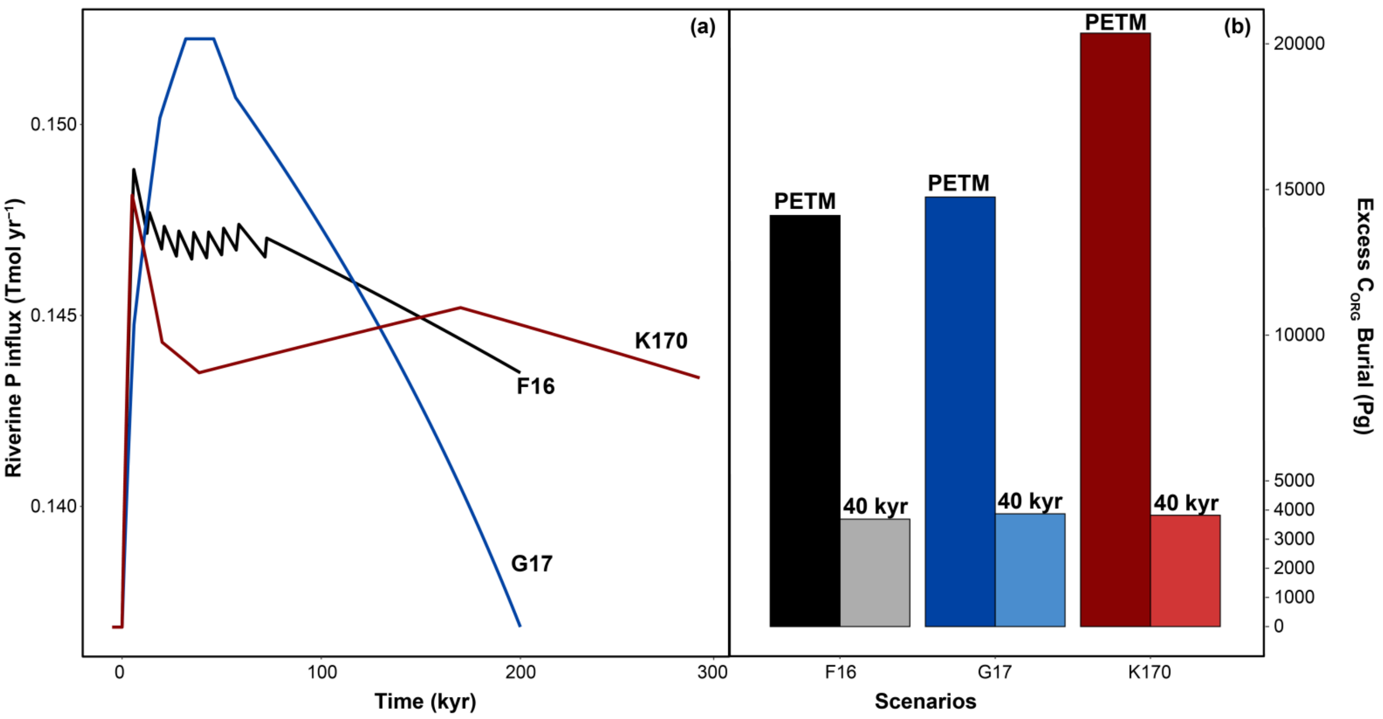

Figure S3. Overview of two key input and output parameters for three additional PETM scenarios. The increased riverine phosphorus flux (A) is forced using pCO_2 curves from Frieling *et al*., 2016 (F16; black), Gutjahr *et al*., 2017 (G17; blue) and Zeebe *et al*., 2009, with the longer plateau duration of Zeebe and Lourens, 2019 (K170; red). Darker colors for excess organic carbon (C_{org}) burial (B) indicate burial calculated over the entire PETM interval. Lighter colors indicate burial for the first 40 kyr of the recovery.

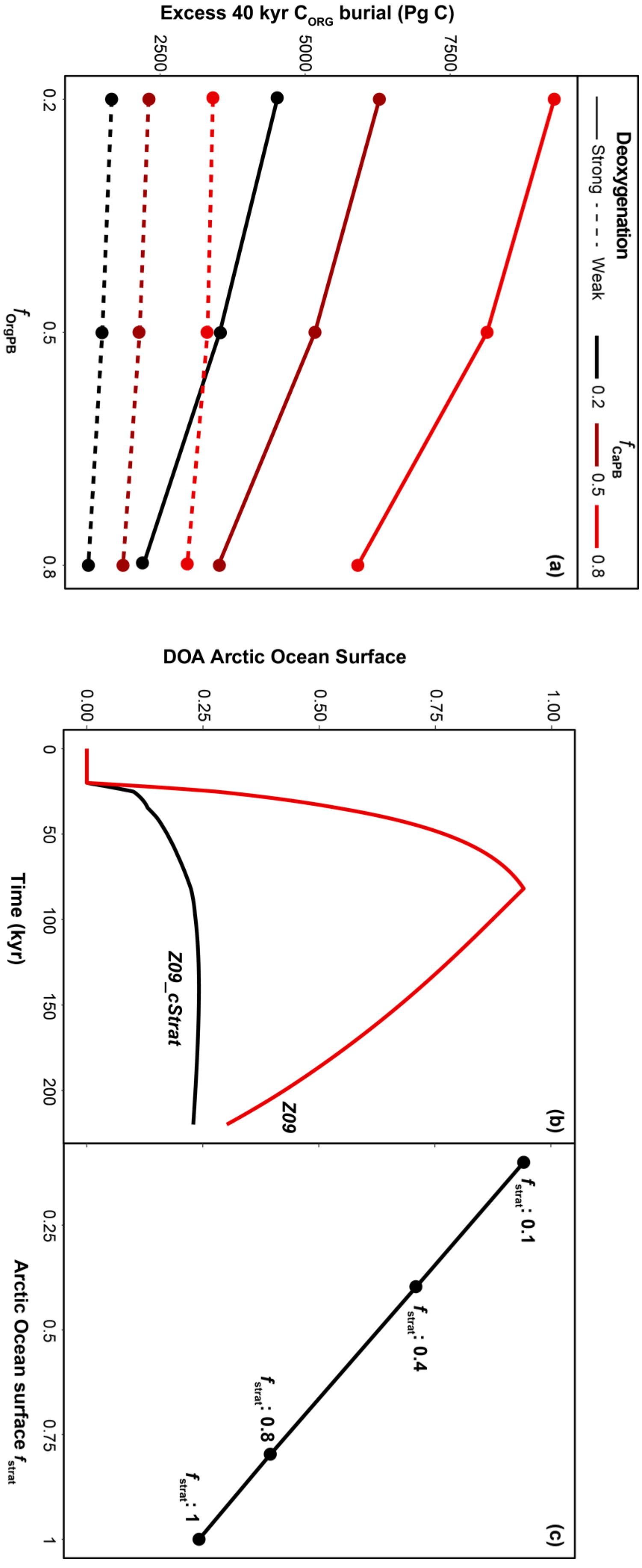
Excess 40 kyr C_ORG burial (Pg C)
2500
5000
7500
Deoxygenation
Strong
Weak
f_CaPB
0.2
0.5
0.8
(a)
f_OrgPB
DOA Arctic Ocean Surface
0.00
0.25
0.50
0.75
1.00
(b)
Z09
Z09_cStrat
Time (kyr)
0
50
100
150
200
(c)
f_strat: 0.1
f_strat: 0.4
f_strat: 0.8
f_strat: 1
Arctic Ocean surface f_strat
0.25
0.5
0.75
1

Figure S4. Overview of sensitivity tests performed for our carbon-cycle box model. The effect of increased redox sensitivity of P burial on excess C_{org} burial within the first 40 kyr of the recovery (A) was tested under strong (high Degree of Anoxia or DOA; solid line) and weak deoxygenation (low DOA; dashed line). The values that were tested for the redox sensitivity of organic P burial (f_{OrgPB}) are 0.2, 0.5 and 0.8. The same values were tested for the redox sensitivity of authigenic P burial (f_{CaPB}; black, dark red and red, respectively). The effect of constant versus increased stratification (B) and different degrees of increased stratification (C) was tested on the DOA of the surface Arctic Ocean (box S1). Black line: constant stratification (Z09_cStrat). Red line: increased stratification with a maximum value of f_{strat} = 0.1 (Z09).

Table ST1. (Uploaded separately) Compilation of published and new proxy records. Records cover proxies that are used to reconstruct changes in primary productivity and redox conditions. Site numbers correspond to the ones used in Figs. 4 and 5, and all references for these figures are given here as well. The key explains symbols and letters used to indicate increased, decreased or constant productivity and deoxygenation. We also provide information on the timing of these changes relative to the following stages of the $\delta^{13}C$ excursion: onset (O), core (B), early recovery (Ta) and late recovery (Tb). For an overview of these phases, see Fig. 1 of the main text. For changes in redox conditions we also provide the interpretations given in the original studies. Complete references are given at the end of the main text.

Table ST2. Descriptions for the standard runs and sensitivity simulations performed within this study. An np value of 0.4 results in a maximum riverine P input rate of ~0.15 Tmol P yr^{-1} and a value of 1.0 gives a maximum rate of ~0.17 Tmol P yr^{-1}. Note that the following sensitivity analyses were also performed with an np value of 1.0: Z09_OOP, Z09_nostrat, Z09_noO2r, Z09_noPPr and Z09_river.

Code	CO_2 Forcing	f_{OrgP}[a]	f_{CaP}[a]	f_{strat}[a]	n_p[a]	O_2 dependence	Notes
Z09	Zeebe *et al.*, 2009	0. 75	0.4	0.1[b]; 1[c]; 0.4[d]	0.4	T; f_{strat}; PP	
Z09_weath				0.1[b]; 1[c]; 0.4[d]	**1**	T; f_{strat}; PP	Increased P weathering sensitivity
Z09_OOP				0.1[b]; 1[c]; 0.4[d]	0.4	T; f_{strat}; PP	PP in S4 and S5 constant
Z09_nostrat				**1[b]; 1[c]; 1[d]**	0.4	T; PP	No stratification
Z09_river				**1[b]; 1[c]; 1[d]**	0.4	-	No deoxygenation/ stratification
Z09_O_2sol				**1[b]; 1[c]; 1[d]**	**0**	**T; PP**	No P weathering/ stratification
F16	Frieling *et al.*, 2016	0.75	0.4	0.1[b]; 1[c]; 0.4[d]	0.4	T; f_{strat}; PP	Supp. Info.
G17	Gutjahr *et al.*, 2017						
K170	Zeebe *et al.*, 2009 + 170 kyr body						

a: Sensitivity analysis in Supplementary Information; b: Arctic surface; c: Open ocean continental shelf; d: European Epicontinental shelf surface

Table ST3. (Uploaded separately) Maximum and minimum (O_2 concentration) values for key model output, and excess C_{org} burial over the entire PETM and the first 40 kyr of the recovery, for our standard scenario and further sensitivity simulations. An explanation for the setup of the sensitivity analyses can be found in Table 4. The change in temperature is the same for all scenarios and given only for the standard run (Z09).

Table ST4. (Uploaded separately) Box characteristics with steady state concentrations and fluxes. Fluxes for carbon are given in Pg C yr^{-1}, for phosphorus in Tmol P yr^{-1} and for oxygen in Tmol O_2 yr^{-1}. All values are rounded.

Table ST5. Water fluxes for the box-model. Values are given in Tm^3/yr (model input) and Sverdrup.

Flux	Code	Tm3/yr	Sv
Arctic surface - deep	F1/F2	70	2.2
Arctic surface to EES surface	F3	65	2.1
EES surface to Arctic surface	F4	63	2.0
Arctic surface - mid/low latitudes	F5/F6	43	1.4
EES surface - mid/low latitudes	F7/F8	154	4.9
EES surface -deep	F9/F10	80	2.5
Open ocean shelf to mid/low latitudes	F11	631	20.2
Open ocean shelf upwelling	F12	631	20.2
Mid/low latitudes Upwelling	F13	158	5.0
Mid/low latitudes - thermocline	F14/F15	3345	107.0
Southern ocean - Atlantic	F16/F17	158	5.0
Southern ocean - Indotethys	F18/F19	158	5.0
Southern ocean - Pacific	F20/F21	252	8.1
Southern ocean deep water (Atlantic)	F22	131	4.2
Southern ocean deep water (Indotethys)	F23	202	6.5
Southern ocean deep water (Pacific)	F24	456	14.6
Thermocline upwelling (Atlantic)	F25	131	4.2
Thermocline upwelling (Indotethys)	F26	202	6.5
Thermocline upwelling (Pacific)	F27	456	14.6
Mid/low latitudes to Southern Ocean	F28	394	12.6
Riverine influx (Arctic)	RF1	5	0.2
Riverine influx (EES)	RF2	18	0.6
Riverine influx (Open ocean shelf)	RF3	28	0.9
Evaporation (Arctic)	EV1	3	0.1
Evaporation (EES)	EV2	20	0.6
Evaporation (Open ocean shelf)	EV3	28	0.9
Evaporation (Mid/low latitude)	EV4	394	12.6
Precipitation (Southern Ocean)	PR1	394	12.6

Table ST6. Key parameter values for the box model.

Parameter	Symbol	Value	Unit
Redfield C_{org}/P_{org}	$(C_{org}/P_{org})_{Redfield}$	106	mol/ mol
Redfield C_{org}/O_2	$(C_{org}/O_2)_{Redfield}$	106/138	mol/ mol
Fraction of export production decayed in thermocline	*tcpfract*	0.78	-
Surface particulate OM export fraction	f_{exp}	0.15[S1]; 0.03[S2]; 0.04[S3]; 0.08[S4]; 0.1[S5]	-
Fraction P_{riv} input	f_{in}	0.1[S1]; 0.55[S2]; 0.35[S3]	-
Anoxic C_{org}/P_{org}	$(C_{org}/P_{org})_{anoxic}$	1500	mol/ mol
Burial C_{org}/P_{org}	$(C_{org}/P_{org})_{oxic}$	400[S1,S3]; 500[S2]; 260[D1-5]	mol/ mol
Rate constant for primary productivity	k_{biol}	0.86[S1]; 0.99[S2]; 0.99[S3]; 0.8[S4]; 0.51[S5]	Tm^3/yr
Fraction primary/export productivity buried (P_{org})	k_{OrgP}	0.0039[S1]; 0.0028[S2]; 0.0045[S3]; 0.0041[D1,D5]; 0.0007[D2]; 0.001[D3]; 0.0024[D4]	-
Fraction decay buried (P_{auth})	k_{CaP}	0.009[S1,S3]; 0.006[S2]; 0.008[D1,D5]; 0.031[D2-4]	-
Steady state temperature	*T0*	17[S1]; 25[S2-4]; 12[S5]; 16[IM]; 12[D1-5]	˚C
Gravimetric constants A	*A*	-177.7888; 255.5907; 146.4813; -22.2040	-
Gravimetric constants B	*B*	-0.037362; 0.016504; -0.0020564	-
Silicate weathering scaling factor	n_s	0.2	-
Carbonate weathering scaling factor	n_{cc}	0.4	-
Surface waters temperature relaxation time	*tlags*	20	-
Intermediate waters temperature relaxation time	*tlagim*	200	-
Deep waters temperature relaxation time	*tlagd*	1000	-
Temperature sensitivity to doubling of CO_2	*s*	3	-
Salinity	*Sal*	34.72	(psu)

Table ST7. (Uploaded separately) Box characteristics with steady state concentrations and fluxes. Fluxes for carbon are given in Pg C yr^{-1}, for phosphorus in Tmol P yr^{-1} and for oxygen in Tmol O_2 yr^{-1}. All values are rounded.

Table ST8. (Uploaded separately) Calculations of sedimentation rates and organic carbon mass accumulation rates for locations with TOC records. An explanation for the calculations, and the dry bulk density used in each case, can be found in the Supplementary Text.

Data Set S1. Core information and new bulk geochemical measurements for study sites presented in this study and its supplements. The new data presented here cover: total organic carbon content (C_{org}), aluminum (Al), iron (Fe), manganese (Mn), molybdenum (Mo), phosphorus (P), vanadium (V). Data also available on PANGAEA.

PANGAEA Data DOI Information:

Papadomanolaki, NM; Sluijs, A; Slomp, CP (2021): Carbonate geochemistry of ODP Site 174AX (Bass River): https://doi.pangaea.de/10.1594/PANGAEA.929260

Papadomanolaki, NM; Sluijs, A; Slomp, CP (2021): Major element composition of ODP Site 174AX (Bass River): https://doi.pangaea.de/10.1594/PANGAEA.929259

Papadomanolaki, NM; Sluijs, A; Slomp, CP (2021): Carbonate geochemistry of IODP Hole 302-M0004A: https://doi.pangaea.de/10.1594/PANGAEA.929258

Papadomanolaki, NM; Sluijs, A; Slomp, CP (2021): Major element composition of IODP Hole 302-M0004A: https://doi.pangaea.de/10.1594/PANGAEA.929255

Papadomanolaki, NM; Sluijs, A; Slomp, CP (2021): Carbonate geochemistry from an outcrop sample at Forada Site, Southern Alps, Italy: https://doi.pangaea.de/10.1594/PANGAEA.929262

Papadomanolaki, NM; Sluijs, A; Slomp, CP (2021): Major element composition from an outcrop sample at Forada Site, Southern Alps, Italy: https://doi.pangaea.de/10.1594/PANGAEA.929261

Papadomanolaki, NM; Sluijs, A; Slomp, CP (2021): Carbonate geochemistry of ODP Hole 121-752A: https://doi.pangaea.de/10.1594/PANGAEA.929304

Papadomanolaki, NM; Sluijs, A; Slomp, CP (2021): Major element composition of ODP Hole 121-752A: https://doi.pangaea.de/10.1594/PANGAEA.929303

Papadomanolaki, NM; Sluijs, A; Slomp, CP (2021): Carbonate geochemistry of IODP Hole 342-U1403A: https://doi.pangaea.de/10.1594/PANGAEA.929264

Papadomanolaki, NM; Sluijs, A; Slomp, CP (2021): Major element composition of IODP Hole 342-U1403A: https://doi.pangaea.de/10.1594/PANGAEA.929263

Papadomanolaki, NM; Sluijs, A; Slomp, CP (2021): Major element composition of ODP Hole 189-1172D: https://doi.pangaea.de/10.1594/PANGAEA.929305

Papadomanolaki, NM; Sluijs, A; Slomp, CP (2021): Major element composition from an outcrop sample at Lodo Gulch Site, Panoche Hills, California: https://doi.pangaea.de/10.1594/PANGAEA.929308

Papadomanolaki, NM; Sluijs, A; Slomp, CP (2021): Carbonate geochemistry of ODP Hole 189-1172D: https://doi.pangaea.de/10.1594/PANGAEA.929307
