# Supplementary material for: Eutrophication and Deoxygenation Forcing of Marginal Marine Organic Carbon Burial During the PETM
Source: Paleoceanogr Paleoclimatol. 2022 Mar 3;37(3):e2021PA004232. doi: 10.1029/2021PA004232 (PMC9310739; doi:10.1029/2021PA004232)
Supplement: Supplementary file 2 — Table S1 [file PALO-37-0-s002.pdf]

No.	Site	Setting/ Paleodepth	Primary Productivity				Oxygen					Author oxygen condition definition	C_{ORG} max. concentrations (%)	C_{ORG} refs.
			Change	Data	Timing	Reference	Change	Data	Timing	Reference	Max. (average) Mo values (ppm)			
1	Harrell Core, Gulf Coastal Plain	Shelf					~	Isorenieratane	B (1/2)	Sluijs et al., 2014		Seasonal photic zone euxinia	~1.6	Sluijs et al., 2014
								Foram linings						
								Glauconite						
2	Wilson Lake, New Jersey Shelf	Shelf	**+**	Nannofossils	O-Ta (/2)	Gibbs et al., 2006	**-**	Benthic forams	O-Ta (/ 2)	Stassen et al., 2012		Dysoxic (biogroups)	~0.7	Lippert & Zachos, 2007
			**+?**	Benthic forams	O-Ta (/2)	Stassen et al., 2012; 2015	**-**	Biogenic single domain magnetite	O-B (/ 2)	Lippert & Zachos, 2007		Non-steady state redox/ Oxic-anoxic trans. shifted		
3	Ancora, New Jersey Shelf	Shelf	**+**	Benthic forams	O-Tb (/2)	Stassen et al., 2012	**-**	Benthic forams	O-Ta (/ 2)	Stassen et al., 2012		Dysoxic (biogroups)		-
							**-**	Biogenic single domain magnetite	O-Ta (/ 2)	Stassen et al., 2012		Non-steady state redox/ Oxic-anoxic trans. shifted		
4	Bass River	Shelf	**+**	Benthic forams	O-Tb (/2)	Stassen et al., 2012; 2015	**-**	Benthic forams	O-Ta (/ 2)	Stassen et al., 2012		Dysoxic (biogroups)	~0.8	This study
								Biogenic single domain magnetite	Ta (?)				1.2	John et al.. 2008
							**=/~**	Trace elements	O-Tb (/ 2)	This study	1.69 (0.32)			
5	Lodo Gulch	Shelf					**=/~**	Trace elements	O-Tb	This study	0		~1.8	John et al.. 2008
6	IODP Site M0004, Lomonosov Ridge	Shelf	**+**	Organic carbon composition	O-T (low resol though)	Stein et al., 2006	**-**	Isorenieratane	B-Ta	Sluijs et al., 2006		Photic zone euxinia	3.8	This study
								Redox-sensitive trace elements	B-Ta (/)	Sluijs et al., 2008		Anoxia		
								Laminations						
								Foram linings						
								Pyrite	B-Ta	Stein et al., 2006		Euxinic		
							**-/~**	Redox-sensitive trace elements	B/Ta	This study	105.33 (26.74)			
	[illegible]							Amorphous organic matter						

7	Longyearbyean, Spitsbergen	Shelf					~	Phytoclasts	T	Harding et al., 2011		(Seasonally) low oxygen	~2.5	Harding et al., 2011
								Th/U						
								Laminations						
8	BH9-05, Spitsbergen	Shelf					-	Benthic forams	?	Nagy et al., 2013		Hypoxia	~4	Cui et al., 2011
								Laminations						
								Laminations	?	Dypvik et al., 2011		Reducing conditions		
								Th/U						
								Pyrite						
								pristane, phytane	O-Ta?	Cui et al., 2011		Anoxia		
9	Central Basin, North Sea	Shelf					-	Laminations	?	Sluijs personal observations (in Sluijs et al., 2014)			~2.8	Kender et al., 2012
			**(=)+**	dinoflagellates/ forams?		Kender et al., 2012		Benthic forams/ amorphous Organic Matter?	O-B	Kender et al., 2012		Anoxia		
10	Denmark, North Sea	Shelf					-	Laminations	O-B	Schoon et al., 2015		Anoxic/sulfidic	~4.5	Schoon et al., 2015
								Pyrite						
								Isorenieratane						
11	Well 10, West Siberian Sea	Epicontinental Shelf					~	Isorenieratane	? (1/)	Frieling et al., 2014		Photic zone euxinia	~8	Frieling et al., 2014
								total organic carbon				Intermi./seas. Anoxia		
								Dinoflagellates						
12	Medani, Georgia	Epicontinental Shelf					-	Redox-sensitive trace elements	? No d13C (B)	Gavrilov et al., 1997; 2003	~10	Hypoxic?	~4	Gavrilov et al., 1997
13	Kheu River, Central Caucasus	Epicontinental Shelf	**+**	Pristane; Phytane	B	Dickson et al., 2014	-	Redox-sensitive trace elements	? No d13C (B)	Gavrilov et al., 1997; 2003	635		~9	Dickson *et al.*, 2014
				Alkanes										
				Nannofossils/ dinoflagellates		Scherbinina et al., 2016		Redox-sensitive trace elements	O-Ta	Dickson et al., 2014	>1110	Anoxic/episodic ventilation	~8	Gavrilov et al., 1997
	Kaurtakapy	Epicontinental						Pyrite/ fish debris		Bolle et al., 2000		Low oxygen conditions		

14	Kaurtakapy, Khazakstan	Epicontinental Shelf					-	Pyrite	?	Gavrilov et al., 1997; 2003				-
15	Aktumsuk, Uzbekistan	Epicontinental Shelf					-	Trace metals	? No d13C (B)	Gavrilov et al., 1997; 2003	~8	Hypoxic?	~5	Gavrilov et al., 1997
							=-	laminations	?	Gavrilov et al., 2003				
							-	Pyrite/ lycopane	?	Bolle et al., 2000		Anoxic		
16	Torangly, Turkmenistan	Epicontinental Shelf	+	Benthic forams	B	Speijer et al., 1997	-	Benthic forams	B	Speijer et al., 1997		Anaerobic/ dysaerobic	~2.5	Gavrilov et al., 1997
								Redox-sensitive trace elements	? No d13C (B)	Gavrilov et al., 1997; 2003	360			
17	Dzenghutay	Epicontinental Shelf					~	Redox-sensitive trace elements	B	Dickson et al., 2014	~10	Suboxic	~3.5	Dickson et al., 2014
18	Kurpai / Guru Fatima, Tadjik depression	Epicontinental Shelf					-	Redox-sensitive trace elements	B	Dickson et al., 2014	>100	Anoxia/euxinia	~15	Gavrilov et al., 2003
								Redox-sensitive trace elements	? No d13C (B)	Gavrilov et al., 1997; 2003	188		~20	Dickson et al., 2014
19	Sidi Nasseur / Wadi Mezaz, Tunisia	Shelf	=+	Benthic forams		Stassen et al., 2012	-	Benthic forams	O/B-?	Stassen et al., 2012		Severe dysoxia	~0.4	Stassen et al., 2012
				Urchins				Benthic forams	B	Morsi et al., 2011		Low oxygen		
								Ostracodes						
20	Gebel Aweina, Egypt	Shelf	+	Forams	O-B (/2?)	Speijer & Schmitz, 1998	-	Laminations	?	Speijer & Schmitz, 1998				-
								Forams	O-B (?)			Hypoxic?		
								Benthic forams		Speijer & Wagner, 2002				
21	Dababiya, Egypt	Shelf	+	Phosphatic concretions	?	Ernst et al., 2006	-	Benthic forams (and compl. Absence)	O-Ta	Ernst et al., 2006		Anoxic/ ephemeral oxygenation	~3	Schulte et al., 2011
				Fish remains	?			Laminations						
				Forams	O-T			Redox-sensitive trace elements	B	Soliman et al., 2011		Anoxic/Euxinic		
				Phosphatic debris	?	Soliman et al., 2011		Redox-sensitive trace elements	B	Khozyem et al., 2015	$\sim 50*10^{-4}$ (Mo/Al)	Anoxic		
				Trace elements	B									
				d15N	O-T	Khozyem et al., 2015								
				Elements	T (?)									

22	Gebel Duwi, Egypt	Shelf	+	Forams	?	Speijer et al., 1996	**?**	Forams	?	Speijer et al., 1996			~0.15	Bolle et al., 2000
				Phosphates	O-T	Bolle et al., 2000	-	Laminations	?					
			**?**	Opal	?	Schmitz et al., 1997	-	Forams		Speijer & Wagner, 2002				
				d13C										
				Calcite										
23	Gebel Nezzi, Egypt	Shelf					-	Benthic forams		Soliman et al., 2011				
24	Gebel Qreiya, Egypt	Shelf	+	P dynamics	O-B?	Schulte et al., 2013	-	Benthic forams		Khozyem et al., 2015		Hypoxia? (intermittent?)	~4.5	Schulte et al., 2013
								Pyrite	? (no access)	Knox et al., 2003				
								Redox-sensitive trace elements						
								Pyrite		Soliman, 2003				
								Redox-sensitive trace elements						
								Redox-sensitive trace elements	O-B/Ta	Schulte et al., 2013		Anoxia/brief euxinia		
25	Wadi Nukhul, Egypt	Shelf	+	d15N	O(-T)	Khozyem et al., 2013	-	Benthic forams		Speijer & Wagner, 2002				-
				Elements				Benthic forams	O-B (?)	Speijer et al., 1997		Quasi-anaerobic/ Dysaerobic		
				Phosphorus				Redox-sensitive trace elements	O-T	Khozyem et al., 2013				
				Benthic forams	O-B (?)	Speijer et al., 1997		d15N						
26	Ben Gurion, Israel	Shelf	+	Barium	O-B/Ta? (low resol)	Schmitz et al., 1997	-	Benthic forams		Speijer & Wagner, 2002				-
				SiO_2										
				P_2O_5										
				Radiolaria										
27	ODP Site 1172, Tasman Plateau	Shelf					~	Redox-sensitive trace elements	O-T	This study	2.87 (1.51)		~0.7	This study

28	ODP Site 738	Deep (1300m)	**+ (XP)**	Barite	B? (low resol)	Ma et al., 2014	**=-**	Mn/U enrichment factors		Pälike et al., 2014		Suboxic	0	Shipboard Scientific Party, 1989a
							-	Forams	?	Lu & Keller, 1993				
29	DSDP Site 752, Kerguelen Plateau	Slope					?	Redox-sensitive trace elements	?	This study	0.6 (0.2)		~0.4	This study
30	DSDP 213, Indian Ocean		-	Nannofossil assemblages	O-B (/1)	Tremolada & Bralower, 2004								-
31	Khasi Hills, India		**+**	Ter OM + Apectodinium		Prasad et al., 2006								-
32	TDP14		**=-**	Nannofossil assemblages	O-B	Bown & Pearson, 2009							~0.9	Aze et al., 2014
33	IB10 Nigeria	Shelf	**+**	TOC/Dinoflagellates	O/T	Frieling et al., 2017	-	Redox-sensitive trace elements	O-B	Frieling et al., 2017	~7	(Intermittent) Anoxia/PZE	1.6	Frieling et al., 2017
34	ODP Site 959, West Africa	slope	**+**	Dinoflagellates	B	Frieling et al., 2018	**+(?-)**	Foram linings	B	Frieling et al., 2018		Decreased oxygen levels	~1.4	Frieling et al., 2018
							-	Redox-sensitive trace elements						
35	1051	deep	**+ (XP)**	Barite	B	Ma et al., 2014								-
36	South Dover Bridge		**+**	Nannofossil assemblages	O-B, T?	Self-Trail et al., 2017								-
37	IODP Site 1403, Newfoundland	Deep					**-?**	Redox-sensitive trace elements	O	This study	4.4 (0.5)		~0.3	This study
38	DSDP Site 549, North Atlantic	Deep	**+ (XP)**	Barite	T	Ma et al., 2014							~0.1	Graciansky et al., 1985 (but not PETM specifically)
39	DSDP Site 401, Bay of Biscay	Deep (2000m)	-	Nannofossil assemblages	O-B	Tremolada & Bralower, 2004	-	Mn, U enrichment factors	O-T	Pälike et al., 2014		Suboxic		-
40	Zumaia, Spain	slope/rise					-	Ichnofossils	O-B/Ta	Rodriguez-Tovar et al., 2011		Oxygen depletion (not full anoxia)	~0.6	Dunkley Jones et al., 2018
							-	Forams	?	Canudo et al., 1995		Low oxygen		

41	Alamedilla, Spain	slope/rise	**=?(meso)**	Epi vs infaunal	?	Alegret et al., 2010	**=+**	Vr/Cr Ni/Co		Alegret et al., 2010		Oxic		-
42	Caravaca, Spain	slope 200-600					**- (?)**	Laminations	?	Canudo et al., 1995		Low oxygen		-
43	Forada, Italy	rise/deep	**+**	Nannofossil assemblages	B	Agnini et al., 2007	**=+**	Forams		Giusberti et al., 2007		Oxic	0.6	Giusberti et al., 2007
			**+/=+**	Planktonic forams	O-B	Luciani et al., 2007		Redox-sensitive trace elements						
			**~**	Barite	O	Giusberti et al., 2007		Bioturbation						
			**(=)+ (XP)**		B-Ta		**-**	Forams	O-B	Luciani et al., 2007		Oxygen depletion		
			**+**	Benthic forams	B	Giusberti et al., 2016	**-**	Redox-sensitive trace elements	O	This study	0			
							**-**	Benthic forams	O/B	Giusberti et al., 2016		Brief anoxia		
44	Anthering, Austria	rise	**+**	Diatoms	? (low resol)	Egger et al., 2003	**-**	Benthic forams	? (low resol)	Egger et al., 2003		Oxygen depletion		-
				Radiolaria										
				Dinoflagellates										
45	Untersberg, Austria	rise	**+**	Radiolaria	?	Egger et al., 2005								-
46	Bieszczady Mountains, Poland	rise	**~**	Radiolaria/deep water agglutinated foraminifera	? (no d13C)	Bak et al., 2004	**-**	Deep water agglutinated forams	O-B (?)	Bak et al., 2004		Low oxygen		-
				Barite			**+**		T (?)					
				P_2O_5					(no d13C)					
47	Hokkaido Tokachi district	Deep (?)					**-**	Benthic forams	?	Kaiho, 1988				-
48	ODP Site 1209, Shatsky Rise; Central Pacific	Deep (2000m)	**-/=-**	Nannofossil assemblages	O-B	Gibbs et al., 2006	**=+**	Mn, U enrichment factors		Pälike et al., 2014		Oxic		-
			**-/=-**	Planktonic forams	?	Petrizzo, 2007	**-**	Benthic forams	O-Ta	Takeda & Kaiho, 2007		Low oxygen		
			**+ (XP)**	Barite	? (low resol)	Ma et al., 2014	**-/=-**	Pyrite	O	Colosimo et al., 2005				
			**-**	Benthic forams	O-Ta	Takeda & Kaiho, 2007								
			**=(+)**	Coccolithophore Sr/Ca		Stoll et al., 2007	**-**	Benthic forams	O-B	Kaiho et al., 2006				

49	ODP Site 1210, Shatsky Rise; Central Pacific	Deep (2200m)	**-/=-**	Planktonic forams	?	Petrizzo, 2007	**=-/-**	Pyrite	O	Colosimo et al., 2005				-
							-	Benthic forams	O-B	Kaiho et al. 2006				
			-	Benthic forams	O-Ta(?)	Takeda & Kaiho, 2007	-		O-Ta(?)	Takeda & Kaiho, 2007		Low oxygen		
50	ODP Site 1211, Shatsky Rise; Central Pacific	Deep (2500m)					-	Pyrite	O-B	Colosimo et al., 2005				-
			-	Benthic forams	O-Ta(?)	Takeda & Kaiho, 2007	-	Benthic forams	O-Ta(?)	Takeda & Kaiho, 2007		Low oxygen		
51	ODP Site 1212, Shatsky Rise; Central Pacific	Deep (2300m)					-	Pyrite	O	Colosimo et al., 2005				-
			-	Benthic forams	?	Takeda & Kaiho, 2007	-	Benthic forams	?	Takeda & Kaiho, 2007		Low oxygen		
52	ODP Site 865, Central Pacific	Deep (1100-1300m)	**-/= ?**	Planktonic forams	?	Kelly et al., 1996	**-/=-**	Benthic forams	O-B(T? low resol)	Thomas, 1998		Low oxygen		-
							**- (?)**		?	Bralower et al., 1995				
53	ODP Site 1220, Central Pacific	Deep (3200m)	**+ (XP)**	Barite	? (low resol)	Ma et al., 2014	**-/=-**	Bioturbation	?	Nomura et al., 2005				-
								Benthic forams						
54	ODP Site 1221, Central Pacific	Deep	**+ (XP)**	Barite	O-B?	Ma et al., 2014	**-/=-**	Benthic forams	?	Nomura et al., 2005				-
								Bioturbation						
							**=+**	Mn/U enrichment factors		Pälike et al., 2014		Oxic		
55	ODP Site 1215	deep 3000	**+ (XP)**	Barite	O-B	Ma et al., 2016	-?	Benthic forams	?	Shipboard Scientific Party, 2002				-
56	ODP Site 999, Carribean	Deep (1500-2500m)					-	Laminations	O-Ta(Tb?)	Bralower et al., 1997		Dysoxic/oxygen deficiency		-
57	ODP Site 1001, Carribean	Deep (1500-2500m)	**+(XP?)**	Barite	?	Bains et al., 2000	-	Laminations	O-Ta(Tb?)	Bralower et al., 1997		Dysoxic/oxygen deficiency		-
58	ODP Site 1258, Demerara Rise	Deep (3000m?)	**=(+) (different trend per species**	Coccolithophore Sr/Ca		Stoll et al., 2007	**=-**	Mn/U enrichment factors		Pälike et al., 2014		Suboxic		-
59	ODP Site 1260,	Deep	**-/=-**	Nannofossil assemblages	?	Schneider et al., 2013	-	Laminations	?	Shipboard Scientific				-

59	Demerara Rise	(2500m?)	+	Nannofossil assemblages	T	Mutterlose et al., 2007	-	Laminations	?	Scientific Party, 2004				-
60	DSDP 525, Walvis Ridge	deep	**+ (XP)**	Barite	B-T	Ma et al., 2014								
61	ODP Site 1262, Walvis Ridge	Deep (3600m)					-	Mn/U enrichment factors	O-B	Pälike et al., 2014		Suboxic		-
										Chun et al., 2010				
62	ODP Site 1263, Walvis Ridge	Deep (1500m)	**+ (XP)**	Barite	O-Ta	Ma et al., 2014	=-	Mn/U enrichment factors		Pälike et al., 2014		Suboxic		-
			**?-**	Nannofossil assemblages	?	Raffi & De Bernardi, 2008								
63	ODP Site 1265, Walvis Ridge	deep	**+ (XP)**	Barite	O-Ta	Ma et al., 2014								-
64	ODP Site 1266, Walvis Ridge	Deep (2600)	**+ (XP)**	Barite	T	Ma et al., 2014	-	Mn/U enrichment factors	O-B	Pälike et al., 2014/Chun et al., 2010		Suboxic		-
65	ODP Site 689, Maud Rise	Deep (1100m)					-	Benthic forams	?	Thomas & Shackleton, 1996		Decreased oxygen levels		-
									?	Thomas, 1989		Low oxygen		
66	ODP Site 690, Maud Rise	Deep (1800m)	**+ (XP)**	Barite	B	Ma et al., 2014	-	Mn/U enrichment factors	T	Pälike et al., 2014		Suboxic		-
					O-B (Ta?)	Bains et al., 2000								
					T	Torfstein et al., 2010								
			-	Nannofossil assemblages	O-T(a) (depends on taxon)	Bralower, 2002		Benthic forams	?	Thomas & Shackleton, 1996		Decreased oxygen levels		
			+	Nannofossil assemblages?	B	Kelly et al., 2005								
			-	Planktonic forams	O-B (depends on A. species)	Kelly, 2002			?	Thomas, 1989		Low oxygen		
			+	Sr/Ca	O/B-T	Stoll & Bains, 2003								
						Stoll et al., 2007								

67	Mead Stream, New Zealand	slope 100-1000	**=-/-**	Marl/Carbonates	? (low resol)	Hollis et al., 2005	-	Laminations	O-B (?)	Nicolo et al., 2010		Oxygen depletion		-
68	Dee Stream, New Zealand	slope 100-1000	-	Marl/Carbonates	O?	Hancock et al., 2003	-	Laminations	O-B (?)	Nicolo et al., 2010		Oxygen depletion		-
								Forams	? (no d13C comp)	Hancock et al., 2003		Dysoxia		
69	Tawanui, New Zealand	slope	**+**	Dinoflagellates	? (no d13C comp)	Crouch & Brinkhuis, 2005	-	Benthic Forams	? (no d13C comp)	Kaiho et al., 1996		Suboxic	~0.4	Crouch et al., 2003
			**+**	Nanofossil assemblages	B-T	Crouch et al., 2003								
			**-/=?**	Forams/calcareous nannofossils	? (no d13C comp)	Kaiho et al., 1996								
