# Supplementary material for: Eutrophication and Deoxygenation Forcing of Marginal Marine Organic Carbon Burial During the PETM
Source: Paleoceanogr Paleoclimatol. 2022 Mar 3;37(3):e2021PA004232. doi: 10.1029/2021PA004232 (PMC9310739; doi:10.1029/2021PA004232)
Supplement: Supplementary file 3 — Table S2 [file PALO-37-0-s004.pdf]

**Maximum (minimum*) values**	**S1**	**S2**	**S3**	**S4**	**S5**	**IM**	**D1**	**D2**	**D3**	**D4**	**D5**	**Total**
**Z09**												
Temperature (°C)	19.5	27.5	27.5	27.5	14.5	18.5	14.4	14.4	14.4	14.4	14.4	-
$[PO_4]$ (µmol kg^{-1})	1.38	1.77	1.39	1.24	0.86	2.06	2.90	1.47	1.61	1.72	2.74	-
Primary Productivity (Pg C yr-1)	0.9	8.6	3.4	59.4	3.1	-	-	-	-	-	-	75.4
Primary Productivity increase(g C m^{-2} yr-1)	109.5	114.3	92.1	69.0	33.3	-	-	-	-	-	-	418.1
DOA	0.94	0.38	0.74	-	-	-	-	-	-	-	-	-
$[O_2]$ ((µM)*	-	-	-	-	-	84.0	0.0	164.4	144.5	129.1	0.0	-
C_{org} burial (Pg C yr^{-1})	0.01	0.12	0.06	-	-	-	0.002	0.01	0.01	0.03	0.002	0.25
C_{org} burial (Pg C m^{-2} yr^{-1})	4.35	4.28	4.39	-	-	-	0.65	0.17	0.17	0.17	0.15	14.33
C_{org}/P_{tot} (rel)	2.4	1.4	1.9	-	-	-	4.0	1.3	1.3	1.4	4.0	-
Total C_{org} burial (Pg C yr^{-1})	-	-	-	-	-	-	-	-	-	-	-	45300
Total C_{org} burial recovery 120 kyr (Pg C yr^{-1})	-	-	-	-	-	-	-	-	-	-	-	32863
Total C_{org} burial recovery 40 kyr (Pg C yr^{-1})	-	-	-	-	-	-	-	-	-	-	-	9698
Excess C_{org} burial (Pg C yr^{-1})	698	5871	3223	-	-	-	176	518	805	1844	173	13308
Excess C_{org} burial 40 kyr (Pg C yr^{-1})	170	1453	800	-	-	-	43	129	201	460	44	3300
**Z09_Weath**												
$[PO_4]$ (µmol kg^{-1})	1.62	2.12	1.64	1.48	1.02	2.46	3.41	1.75	1.93	2.06	3.23	-
Primary Productivity (Pg C yr^{-1})	1.1	10.2	4.0	70.8	3.8	-	-	-	-	-	-	89.9
Primary Productivity increase(g C m^{-2} yr^{-1})	162.5	175.6	137.3	105.5	51.0	-	-	-	-	-	-	631.8
DOA	0.95	0.48	0.78	-	-	-	-	-	-	-	-	-
$[O_2]$ ((µM)*	-	-	-	-	-	60.1	0.0	148.0	124.2	105.9	0.0	-
C_{org} burial (Pg C yr^{-1})	0.02	0.14	0.07	-	-	-	0.002	0.01	0.02	0.04	0.002	0.29
C_{org}/P_{tot} (rel)	2.4	1.5	2.0	-	-	-	4.0	1.4	1.5	1.6	4.0	-
Excess C_{org} burial (Pg C yr^{-1})	-	-	-	-	-	-	-	-	-	-	-	21060
Excess C_{org} burial 40 kyr (Pg C yr^{-1})	-	-	-	-	-	-	-	-	-	-	-	5015
**Z09_cOOPP**												
$[PO_4]$ (µmol kg^{-1})	1.75	1.81	1.89	1.65	0.81	2.10	3.69	1.33	1.45	1.55	3.50	-
Primary Productivity (Pg C yr^{-1})	1.2	9.3	4.7	37.7	2.0	-	-	-	-	-	-	55.0
Primary Productivity increase(g C m^{-2} yr^{-1})	220.6	141.4	186.8	0.0	0.0	-	-	-	-	-	-	545.9
DOA	0.95	0.43	0.81	-	-	-	-	-	-	-	-	-
$[O_2]$ ((µM)*	-	-	-	-	-	99.8	0.0	182.1	164.0	151.7	0.0	-
C_{org} burial (Pg C yr^{-1})	0.02	0.12	0.08	-	-	-	0.003	0.01	0.01	0.02	0.003	0.27

C_{org}/P_{tot} (rel)	2.4	1.4	2.1	-	-	-	4.0	1.2	1.2	1.2	4.0	-
Excess C_{org} burial (Pg C yr^{-1})	-	-	-	-	-	-	-	-	-	-	-	16231
Excess C_{org} burial 40 kyr (Pg C yr^{-1})	-	-	-	-	-	-	-	-	-	-	-	3974
**Z09_cStrat**												
$[PO_4]$ (μmol kg^{-1})	1.12	1.47	1.14	1.02	0.70	1.70	2.35	1.20	1.32	1.41	2.23	-
Primary Productivity (Pg C yr-1)	0.7	7.1	2.8	48.9	2.6	-	-	-	-	-	-	62.2
Primary Productivity increase(g C m^{-2} yr-1)	53.1	60.0	45.1	35.8	17.3	-	-	-	-	-	-	211.3
DOA	0.24	0.25	0.24	-	-	-	-	-	-	-	-	-
$[O_2]$ ((μM)*	-	-	-	-	-	105.8	56.8	179.5	163.1	150.4	47.0	-
C_{org} burial (Pg C yr^{-1})	0.01	0.10	0.05	-	-	-	0.001	0.01	0.01	0.03	0.001	0.21
C_{org}/P_{tot} (rel)	1.2	1.2	1.2	-	-	-	1.5	1.2	1.2	1.2	1.6	-
Excess C_{org} burial (Pg C yr^{-1})	-	-	-	-	-	-	-	-	-	-	-	7464
Excess C_{org} burial 40 kyr (Pg C yr^{-1})	-	-	-	-	-	-	-	-	-	-	-	1674
**Z09_Weath_Only**												
$[PO_4]$ (μmol kg^{-1})	0.95	1.24	0.96	0.86	0.59	1.43	1.99	1.01	1.11	1.19	1.91	-
Primary Productivity (Pg C yr-1)	0.6	6.0	2.4	41.3	2.2	-	-	-	-	-	-	52.5
Primary Productivity increase(g C m^{-2} yr-1)	15.88	19.19	13.52	11.60	5.70	-	-	-	-	-	-	65.9
DOA	0	0	0	-	-	-	-	-	-	-	-	-
$[O_2]$ ((μM)*	-	-	-	-	-	135.2	100.7	204.3	191.6	181.8	85.2	-
C_{org} burial (Pg C yr^{-1})	0.01	0.08	0.04	-	-	-	0.001	0.01	0.01	0.03	0.001	0.19
C_{org}/P_{tot} (rel)	1.07	1.07	1.07	-	-	-	1.02	1.02	1.02	1.02	1.02	-
Excess C_{org} burial (Pg C yr^{-1})	-	-	-	-	-	-	-	-	-	-	-	2248
Excess C_{org} burial 40 kyr (Pg C yr^{-1})	-	-	-	-	-	-	-	-	-	-	-	506
**Z09_O_2Sol**												
$[PO_4]$ (μmol kg^{-1})	0.95	1.23	0.96	0.85	0.59	1.42	1.98	1.00	1.10	1.18	1.88	-
Primary Productivity (Pg C yr^{-1})	0.6	6.3	2.4	41.0	2.2	-	-	-	-	-	-	52.1
Primary Productivity increase(g C m^{-2} yr^{-1})	15.2	17.5	12.9	10.5	5.1	-	-	-	-	-	-	61.1
DOA	0.10	0.10	0.10	-	-	-	-	-	-	-	-	-
$[O_2]$ ((μM)*	-	-	-	-	-	120.3	83.0	189.4	175.6	164.9	65.6	-
C_{org} burial (Pg C yr^{-1})	0.01	0.08	0.04	-	-	-	0.001	0.01	0.01	0.02	0.001	0.17
C_{org}/P_{tot} (rel)	1.09	1.08	1.08	-	-	-	1.16	1.07	1.08	1.09	1.18	-
Excess C_{org} burial (Pg C yr^{-1})	-	-	-	-	-	-	-	-	-	-	-	2130
Excess C_{org} burial 40 kyr (Pg C yr^{-1})	-	-	-	-	-	-	-	-	-	-	-	476
