# Supplementary material for: Eutrophication and Deoxygenation Forcing of Marginal Marine Organic Carbon Burial During the PETM
Source: Paleoceanogr Paleoclimatol. 2022 Mar 3;37(3):e2021PA004232. doi: 10.1029/2021PA004232 (PMC9310739; doi:10.1029/2021PA004232)
Supplement: Supplementary file 4 — Table S3 [file PALO-37-0-s001.pdf]

Box	Code	Area (Tm^2)	Depth (m)	[O_2] (μM)*	[PO_4] (μmol kg^{-1})	Temperature (°C)	Prim. Prod.	C_{org} Decay	C_{org} Export	C_{org} Burial	River. [PO_4]	Prim. Prod.	P_{org} Decay	P_{org} Export	P_{org} Burial	P_{auth} Burial	P_{Fe} Burial	O_2 Respir.
**Arctic Ocean (surface)**	S1	3	200	234	0.88	17	0.58[a]	0.48	0.09	0.009	0.014	0.45	0.38	0.07	0.002	0.004	0.002	-
**Open ocean continental shelf**	S2	27	150	203.2	1.14	25	5.51[b]	5.27	0.17	0.08	0.075	4.33	4.19	0.13	0.013	0.026	0.013	-
**Eurasian Epicontinental Seaway (surface)**	S3	13.5	150	203.2	0.89	25	2.19[c]	2.06	0.09	0.039	0.048	1.72	1.64	0.07	0.008	0.016	0.008	-
**Low/Mid latitudes open ocean**	S4	341.1	150	203.2	0.79	25	37.69[d]	34.68	3.02	-	-	29.63	27.26	2.37	-	-	-	-
**Southern ocean**	S5	34.9	200	258.3	0.54	12	1.98[e]	1.78	0.2	-	-	1.56	1.4	0.16	-	-	-	-
**Thermocline**	IM	341.1	900	133.4	1.31	16	-	2.48	0.7	-	-	-	1.95	0.55	-	-	-	269.15
**Arctic Ocean (deep)**	D1	13.5	1040	100.7	1.84	12	-	0.09	-	0.001	-	-	0.07	-	0.0003	0.001	0.0003	9.28
**Atlantic Ocean**	D2	52.35	2940	203.3	0.92	12	-	0.14	-	0.006	-	-	0.12	-	0.002	0.004	0.002	15.62
**Indo-Tethys Ocean**	D3	80.27	2000	190.4	1.01	12	-	0.22	-	0.009	-	-	0.18	-	0.003	0.006	0.003	23.95
**Pacific Ocean**	D4	181.48	3610	180.4	1.1	12	-	0.5	-	0.02	-	-	0.40	-	0.006	0.013	0.006	54.16
**Eurasian Epicontinental Seaway (deep)**	D5	3	750	80.8	1.74	12	-	0.09	-	0.001	-	-	0.07	-	0.0003	0.0006	0.0003	9.4
**Total**	-	-	-	-	-	-	47.95	47.79	4.27	0.16	0.137	37.69	37.66	3.35	0.035	0.071	0.035	390.15

a: twice the modern value (Arrigo and van Dijken, 2011); b: somewhat higher than modern value (Wollast *et al*., 1998); c: tuned to obtain nutrient utilization efficiency <1; d: Wollast *et al*., 1998; e: Arrigo *et al*., 2008

Prim. Prod. : Primary Productivity

O_2 resp. : O_2 Respiration
