# Supplementary material for: Eutrophication and Deoxygenation Forcing of Marginal Marine Organic Carbon Burial During the PETM
Source: Paleoceanogr Paleoclimatol. 2022 Mar 3;37(3):e2021PA004232. doi: 10.1029/2021PA004232 (PMC9310739; doi:10.1029/2021PA004232)
Supplement: Supplementary file 6 — Table S5 [file PALO-37-0-s006.pdf]

Environment	Site	Reference	Sediment thickness	Approx. average TOC	Short PETM: 170 kyr		Long PETM: 270 kyr	
			(m)	(%wt)	Average SR (cm/kyr)	OC MAR (g/cm2/kyr)	Average SR (cm/kyr)	OC MAR (g/cm2/kyr)
Shelf/Slope	Harrell Core	Sluijs et al., 2014	3	0.39	1.76	0.02	1.03	0.01
	Wilson Lake	Lippert and Zachos, 2007	15.5	0.35	9.12	0.09	5.34	0.05
	Bass River	John et al., 2008	10.3	0.55	6.06	0.09	3.55	0.05
				0.52		0.09		0.05
	Lodo Gulch	John et al., 2008	15	0.3	8.82	0.07	5.17	0.04
	Central North Sea basin	Kender et al., 2012	8.5	1.3	5	0.18	2.93	0.1
	Denmark	Schoon et al., 2015	3.5	1.73	2.06	0.1	1.21	0.06
			5.8	2.84	3.41	0.23	2.00	0.14
	Sidi Nasseur	Stassen et al., 2012	6	0.2	3.53	0.02	2.07	0.01
	Dababiya	Schulte et al., 2011	3	1.24	1.76	0.06	1.03	0.03
	Gebel Duwi	Bolle et al., 2000	0.3	0.1	0.18	0.0005	0.1	0.0003
	Gebel Qreiya	Schulte et al., 2013	2	2.53	1.18	0.07	0.69	0.04
	ODP 1172	This study	0.8	0.64	0.47	0.008	0.28	0.005
	DSDP 752	This study	5.92	0.2	3.48	0.02	2.04	0.01
	TDP14	Aze et al., 2014	11	0.32	6.47	0.06	3.79	0.03
	IB10	Frieling et al., 2017	13	0.6	7.65	0.12	4.48	0.07
	ODP 959	Frieling et al., 2018	1.5	0.58	0.88	0.014	0.52	0.008
	Zumaia	Dunkley Jones et al., 2018	4.65	0.2	2.74	0.015	1.60	0.009
	Forada	Giusberti et al., 2007	0.99	0.16	0.58	0.003	0.34	0.001
	Tawanui	Crouch et al., 2003	0.65	0.2	0.38	0.002	0.22	0.001
	**Average**				**3.45**	**0.06**	**2.02**	**0.04**
	**Maximum**				**9.12**	**0.23**	**5.34**	**0.14**
EES	West Siberian Sea	Frieling et al., 2014	0.6	2.86	0.35	0.02	0.21	0.01
	Medani	Gavrilov et al., 1997	2.25	2.5	1.32	0.08	0.78	0.05
	Kheu River	Dickson et al., 2014	0.55	5	0.32	0.04	0.19	0.02
	Aktumsuk	Gavrilov et al., 1997	0.25	2.5	0.15	0.009	0.09	0.005
	Torangly	Gavrilov et al., 1997	0.5	1.5	0.29	0.01	0.29	0.01
	Dzhengutay	Dickson et al., 2014	0.55	3.5	0.32	0.03	0.19	0.02
	Guru Fatima	Dickson et al., 2014	1.6	10.4	0.94	0.23	0.55	0.14
	**Average**				**0.53**	**0.06**	**0.33**	**0.04**
	**Maximum**				**1.32**	**0.23**	**0.78**	**0.14**

**Arctic**	Lomonosov Ridge	This study	8.5	2.3	5	0.28	2.93	0.16
	Longyearbyean	Harding et al., 2011	18	1.5	10.59	0.43	6.21	0.25
	BH9/05	Cui et al., 2011	30.5	1	17.94	0.48	10.52	0.28
	**Average**				**11.18**	**0.40**	**6.55**	**0.23**
	**Maximum**				**17.94**	**0.48**	**10.52**	**0.28**
**Deep**	ODP 1403	This study	5.69	0.16	3.35	0.014	1.15	0.005
	**Average**				**3.35**	**0.01**	**1.15**	**0.01**
	**Maximum**				**3.35**	**0.01**	**1.15**	**0.01**
**Total (PETM)**	**Average (Pg)**					**13995**		**10839**
						**26257**		**23077**
**Total (recovery 120 kyr)**	**Average (Pg)**					**9879**		**4485**
						**18534**		**9549**
